# The HDAC inhibitor AR42 interacts with pazopanib to kill trametinib/dabrafenib-resistant melanoma cells *in vitro* and *in vivo*

**DOI:** 10.18632/oncotarget.14829

**Published:** 2017-01-27

**Authors:** Laurence Booth, Jane L. Roberts, Cindy Sander, John Lee, John M. Kirkwood, Andrew Poklepovic, Paul Dent

**Affiliations:** ^1^ Departments of Biochemistry and Molecular Biology, Virginia Commonwealth University, Richmond, VA 23298-0035, USA; ^2^ Department of Medicine, Virginia Commonwealth University, Richmond, VA 23298-0035, USA; ^3^ University of Pittsburgh Cancer Institute Melanoma and Skin Cancer Program, Hillman Cancer Research Pavilion Laboratory L1.32c, Pittsburgh PA 15232, USA; ^4^ Chan Soon-Shiong Institute of Molecular Medicine, Culver City, CA 90232, USA

**Keywords:** autophagy, chaperone, death receptor, ER stress

## Abstract

Studies focused on the killing of activated B-RAF melanoma cells by the histone deacetylase (HDAC) inhibitor AR42. Compared to other tumor cell lines, PDX melanoma isolates were significantly more sensitive to AR42-induced killing. AR42 and the multi-kinase inhibitor pazopanib interacted to activate: an eIF2α–Beclin1 pathway causing autophagosome formation; an eIF2α–DR4/DR5/CD95 pathway; and an eIF2α-dependent reduction in the expression of c-FLIP-s, MCL-1 and BCL-XL. AR42 did not alter basal chaperone activity but increased the ability of pazopanib to inhibit HSP90, HSP70 and GRP78. AR42 and pazopanib caused HSP90/HSP70 dissociation from RAF-1 and B-RAF that resulted in reduced ‘RAF’ expression. The drug combination activated a DNA-damage-ATM-AMPK pathway that was associated with: NFκB activation; reduced mTOR S2448 and ULK-1 S757 phosphorylation; and increased ULK-1 S317 and ATG13 S318 phosphorylation. Knock down of PERK, eIF2α, Beclin1, ATG5 or AMPKα, or expression of IκB S32A S36A, ca-mTOR or TRX, reduced cell killing. AR42, via lysosomal degradation, reduced the protein expression of HDACs 2/5/6/10/11. *In vivo*, a 3-day exposure of dabrafenib/trametinib resistant melanoma cells to the AR42 pazopanib combination reduced tumor growth and enhanced survival from ∼25 to ∼40 days. Tumor cells that had adapted through therapy exhibited elevated HGF expression and the c-MET inhibitor crizotinib enhanced AR42 pazopanib lethality in this evolved drug-resistant population.

## INTRODUCTION

Prior studies have demonstrated that histone deacetylase inhibitors such as valproate, vorinostat and AR42 enhance the cytotoxic potential of multi-kinase inhibitors such as sorafenib, regorafenib and pazopanib [1–6]. Several clinical trials using HDAC inhibitors, including AR42, in combination with multi-kinase inhibitors are presently open at VCU Massey Cancer Center (NCT02349867; NCT01075113; NCT02795819; NCT01817751).

Sorafenib and pazopanib, in addition to being inhibitors of protein kinases, were also recently discovered to be potent inhibitors of chaperone ATPase activities. ATPase inhibition was associated with conformational changes in the ATP binding NH_2_-termini of the chaperone proteins and with the abilities of these chaperones to associate and co-localize with other chaperones and client proteins [7, 8]. It has also been reported that acetylation can also alter the activity of chaperones, generally thought to be an inhibitory effect, with the regulatory acetylation of HSP90 controlled by HDAC6 [9, 10]. Recently we determined that that phosphodiesterase 5 inhibitors such as sildenafil (Viagra^®^), via PKG signaling, did not significantly alter basal chaperone ATPase activities but instead facilitated sorafenib and pazopanib to cause further inhibition of chaperone ATPase activities and a more rapid NH_2_-terminus conformational change [11]. The changes in chaperone ATPase activity and conformation after [sorafenib + sildenafil] and [pazopanib + sildenafil] exposure was also reflected in the phosphorylation/activity of key chaperoned proteins [7, 8, 11]. Whether chaperone acetylation also facilitates the chaperone inhibitory effects of a multi-kinase inhibitor is unknown.

There are several classes of histone deacetylase inhibitors: class I (HDACs 1, 2, 3 and 8); class II (HDACs 4, 5, 6, 7, 9 and 10); class IV (HDAC11). The HDAC inhibitor AR42 is an orally bioavailable, phenyl-butyrate-based small molecule, and is a broad spectrum inhibitor of class I and class II histone deacetylase (HDAC) enzymes. HDAC inhibitors are a class of anti-cancer agents that suppress tumor cell growth via a broad spectrum of mechanisms including the ability to induce growth arrest, differentiation, and apoptosis in cancer cells [12–17]. AR42 has been studied in several laboratory-based cancer models including prostate, hepatocellular, ovarian, lymphocytic leukemia, mantle cell leukemia and acute myelogenous leukemia [15, 18]. Safety studies to assess potential adverse effects of AR42 on the central nervous system, the cardiovascular system, and the respiratory system have been completed and no adverse side effects were observed [18]. Two clinical studies have been conducted with AR42. To date, a total of 57 patients have been treated with AR42 in these 2 clinical trials. The RP2D of AR42 for solid tumor patients was 60 mg QD Monday/Wednesday/Friday for three weeks followed by a vacation week. As a single agent, the HDAC inhibitor panobinostat did no display anti-melanoma activity in patients [19].

Because: a) pazopanib is a potent chaperone inhibitor; b) the [pazopanib + AR42] drug combination has pre-clinical and clinical activity in sarcoma and renal carcinoma; and c) that B-RAF and RAF-1 rely on the chaperones HSP90 and HSP70 to maintain their active conformations, we performed studies in multiple PDX models of human melanoma, including vemurafenib resistant isolates, where cells expressed various mutated active forms of B-RAF. Our present pre-clinical findings argue that AR42 may have single agent activity against mutant B-RAF melanoma, and that AR42 facilitates tumor cell killing by the multi-kinase inhibitor pazopanib *in vitro* and *in vivo*.

## RESULTS

**Figure 1:**
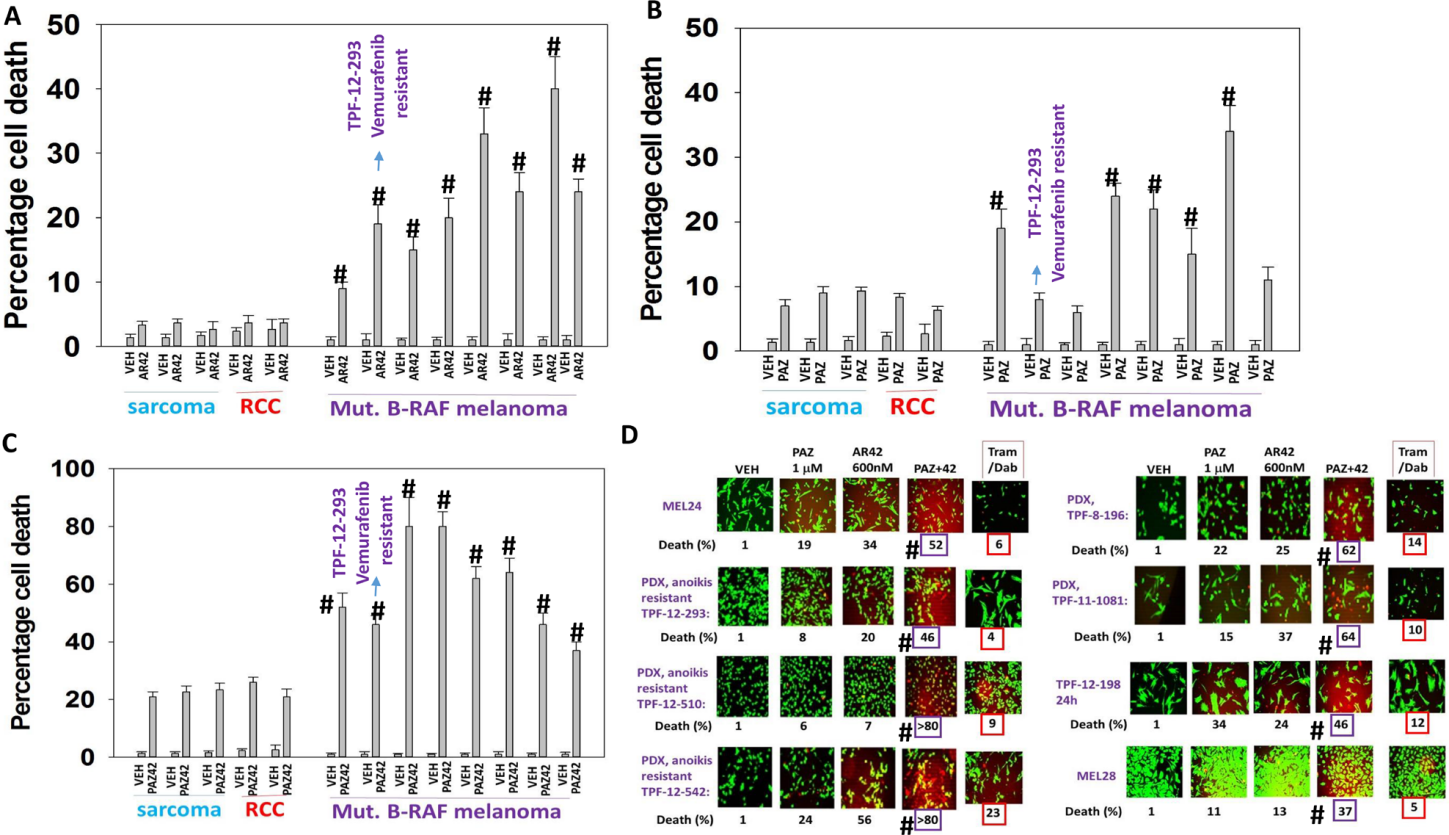
Pazopanib and AR42 interact to kill PDX isolates of mutant B-RAF melanoma including vemurafenib resistant cells (**A**–**C**) Sarcoma cells (HT1080, MES-SA, SKE-S1), renal carcinoma cells (A498, UOK121LN) and B-RAF melanoma cells (SKMEL24, SKMEL28, TPF-12-293, TPF-12-510, TPF-12-542, TPF-8-196, TPF-11-1081, TPF-12-198) were treated with drugs as indicated for 24 h to determine viability (*n* = 3 +/– SEM). ^#^*p* < 0.05 greater than all values in sarcoma and RCCs. (**D**) Melanoma cells were treated with drugs as indicated for 24 h to determined viability. (*n* = 3 +/– SEM). ^#^*p* < 0.05 greater than value in trametinib/dabrafenib treated cells.

AR42, at approximately 40% of its plasma C max, killed mutant B-RAF melanoma cells more effectively than it did sarcoma and renal carcinoma cells (Figure 1A). Pazopanib, at approximately 50% of its free plasma C max, killed mutant B-RAF melanoma cells more effectively than it did sarcoma and renal carcinoma cells (Figure 1B). The combination of [pazopanib + AR42] was also more effective at killing melanoma cells than sarcoma and kidney cancer cells (Figure 1C). The standard of care treatment for mutant B-RAF melanoma is the combination of the MEK1/2/5 inhibitor trametinib and the mutant B-RAF specific inhibitor dabrafenib. In PDX models of mutant B-RAF melanoma AR42 and pazopanib, as single agents, and in combination were more effective at killing the melanoma cells than the combination of trametinib/dabrafenib at their individual 100% C max plasma concentrations (Figure 1D). The TPF-12-293 isolate is vemurafenib resistant and in these cells both AR42 and [pazopanib + AR42] caused high levels of cell killing whereas treatment with trametinib/dabrafenib did not significantly enhance cell death. Similar cell killing data to that in human melanoma cells was obtained in mouse melanoma cells with no known specific driving oncogene addiction, ovarian cancer cells, NSCLC cells, squamous head & neck cancer cells, triple negative breast cancer cells and bladder cancer cells (Supplementary Figure 1A–1F). Similar cell killing data to that obtained using AR42 alone or in combination with pazopanib in melanoma and ovarian cancer cells were also obtained using the generic pan-HDAC inhibitor sodium valproate (Supplementary Figure 2).

**Figure 2:**
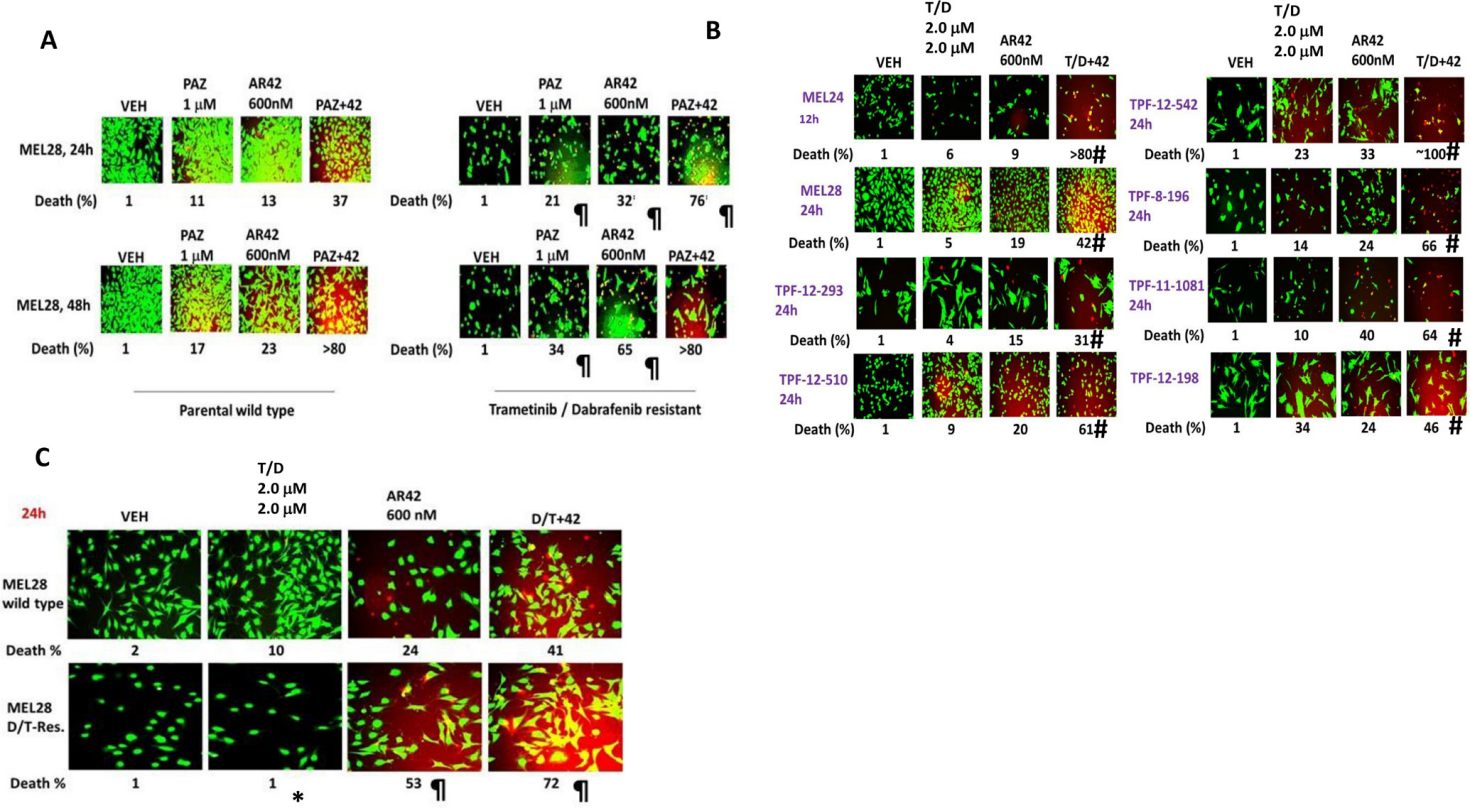
[Pazopanib + AR42] kills trametinib/dabrafenib resistant melanoma cells and re-sensitizes resistant cells to the MEK/B-RAF inhibitor drug combination (**A**) Wild type parental MEL28 and MEL28-R cells were treated with drugs for 24 h to determine viability. (*n* = 3 +/– SEM). ^¶^*p* < 0.05 greater than corresponding value in parental wild type MEL28 cells. (**B**) Cells were treated as indicated with drugs, alone or in in combination, for 24 h to determine viability. (*n* = 3 +/–SEM) ^#^*p* < 0.05 greater than value in T/D treated cells. (**C**) Wild type parental MEL28 cells and MEL28-R cells were treated as indicated with drugs, alone or in combination, for 24 h to determine viability. (*n* = 3 +/– SEM). *p < 0.05 less than corresponding value in parental wild type cells; ^¶^*p* < 0.05 greater than corresponding value in parental wild type cells.

In parallel to the initial studies in Figure 1, we generated trametinib/dabrafenib resistant MEL28 cells (MEL28-R). AR42, pazopanib and the drug combination more effectively killed MEL28-R cells than it did wild type parental cells (Figure 2A). As AR42 was enhancing the anti-cancer properties of a multi-kinase inhibitor, we determined whether it could also enhance trametinib/dabrafenib-induced killing; AR42 and trametinib/dabrafenib interacted in a greater than additive fashion to kill multiple PDX melanoma isolates (Figure 2B). Furthermore, low concentrations of AR42 restored the ability of trametinib/dabrafenib to kill MEL28-R cells (Figure 2C). Collectively, our data demonstrate that pazopanib lethality in a wide range of tumor cell types can be enhanced by HDAC inhibitors.

**Figure 3:**
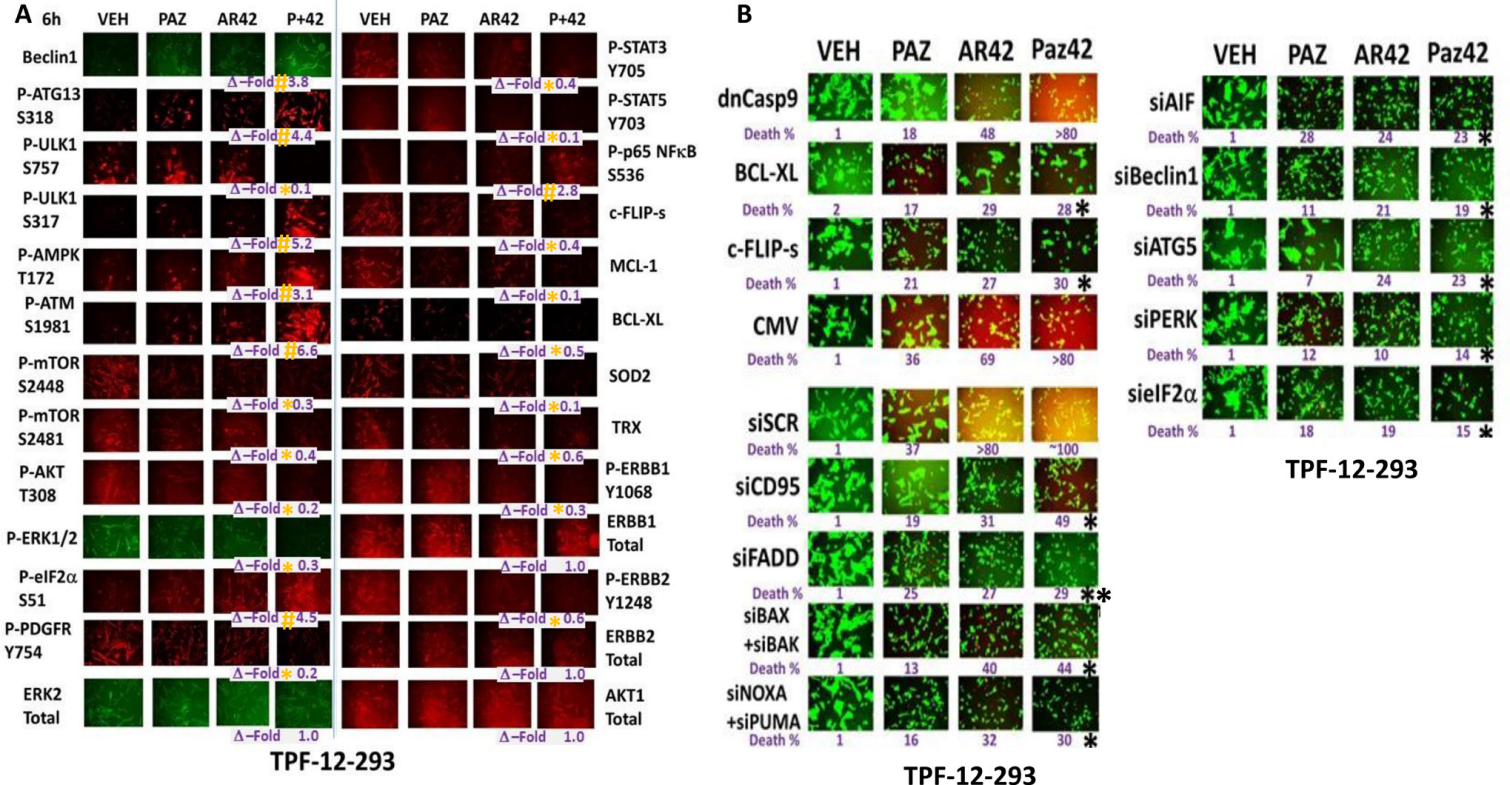
The regulation of protein phosphorylation by [pazopanib + AR42] in mutant B-RAF melanoma cells (**A**) TPF-12-293 vemurafenib resistant cells were treated with drugs for 6 h. Immuno-fluorescence was performed (*n* = 3 +/– SEM). ^#^*p* < 0.05 greater than vehicle control; *p < 0.05 less than vehicle control. (**B**) TPF-12-293 cells were either: transfected with an empty vector plasmid (CMV) or with plasmids to express dominant negative caspase 9, BCL-XL or c-FLIP-s; or with a scrambled siRNA (siSCR) or with siRNA molecules to knock down the indicated proteins. Twenty-four h after transfection cells were treated drugs for 24 h to determine viability. (*n* = 3 +/– SEM). ^¶^*p* < 0.05 greater than corresponding value in siSCR transfected cells; *p < 0.05 less than corresponding value in siSCR/CMV cells **p < 0.05 less than corresponding value in siCD95 cells.

In TPF-12-293 vemurafenib resistant cells, using unbiased screening we discovered that treatment with [pazopanib + AR42] increased the expression of Beclin1 and decreased the expression of c-FLIP-s, MCL-1, BCL-XL, SOD2 and TRX (Figure 3A). *In vitro*, within 6 h, [pazopanib + AR42] exposure increased the phosphorylation of ATG13 S318, ULK1 S317, AMPK T172, ATM S1981, eIF2α S51, p65 NFκB S536; and decreased the phosphorylation of ULK1 S757, mTOR S2448, mTOR S2481, AKT T308, ERK1/2, STAT3 Y705, STAT5 Y703, as well as the phosphorylation of the growth factor receptors ERBB1, ERBB2 and PDGFRβ (Figure 3A). Similar data were obtained in TPF-08-196 cells (Supplementary Figure 3). We next performed additional unbiased screening studies to determine the survival/death -regulatory pathways by which [pazopanib + AR42] killed melanoma cells. Expression of c-FLIP-s and BCL-XL reduced cell killing whereas expression of dominant negative caspase 9 was marginally effective (Figure 3B). Knock down of the death receptor CD95 and to a greater extent the adaptor protein FADD suppressed cell killing. Knock down of [BAX+BAK], [NOXA+PUMA], AIF, Beclin1, ATG5, PERK or eIF2α reduced cell killing. Similar data were obtained in TPF-12-510 and MEL28-R isolates (Supplementary Figure 4A and 4B).

**Figure 4:**
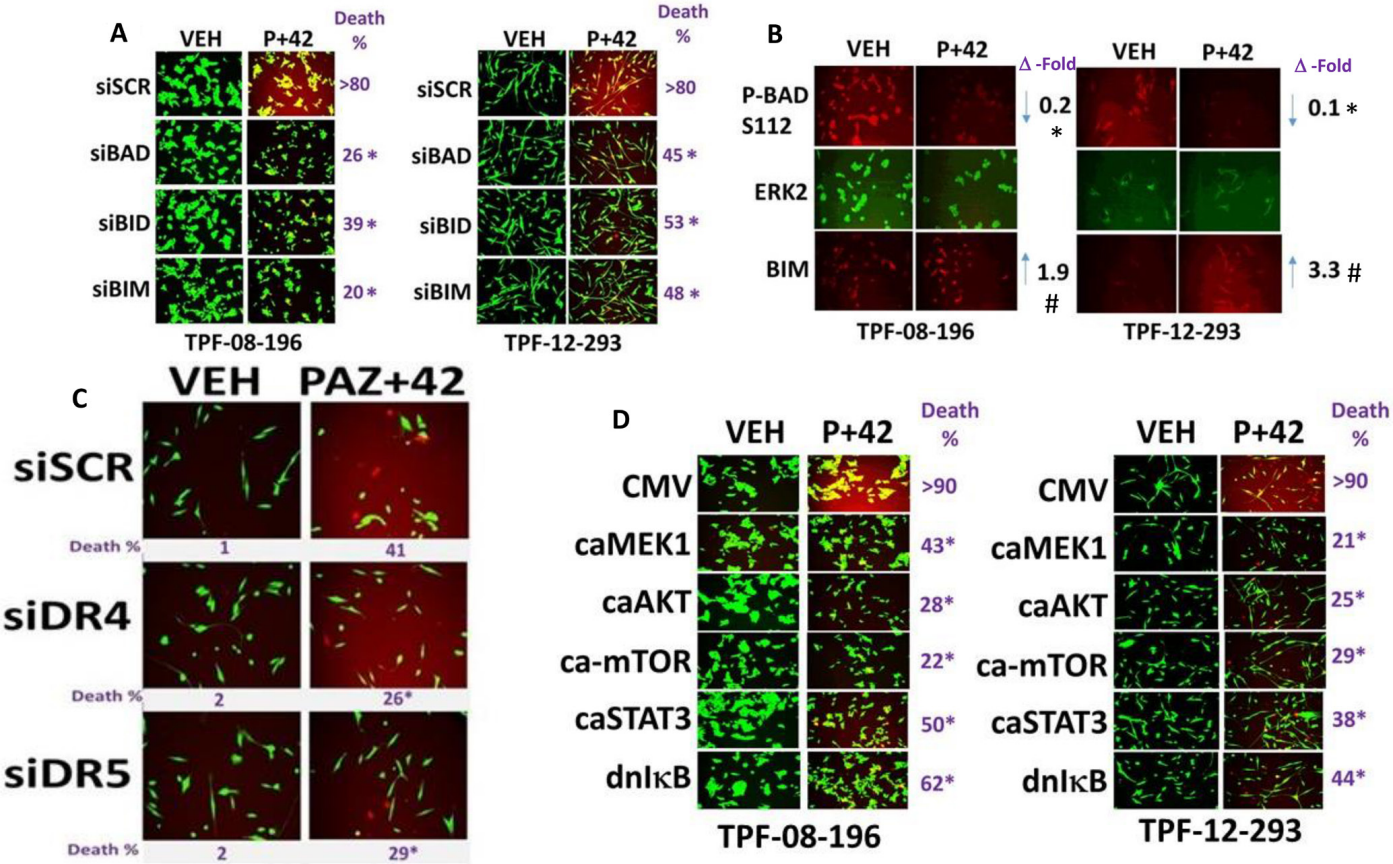
Multiple toxic BH3 domain proteins are required to mediate the death response to [pazopanib + AR42] (**A**) TPF-12-293 and TPF-08-196 cells were transfected to knock down the indicated proteins. Twenty-four h after transfection cells were treated with drugs for 24 h to determine viability. (*n* = 3 +/– SEM). *p < 0.05 less than corresponding value in siSCR cells. (**B**) TPF-12-293 and TPF-08-196 cells were treated with drugs for 6 h. Immuno-fluorescence was performed (*n* = 3 +/– SEM) *p < 0.05 less than corresponding value in vehicle control; ^#^*p* < 0.05 greater than corresponding value in vehicle control. (**C**) TPF-12-293 cells were transfected to knock down the expression of DR4 or DR5. Twenty-four h after transfection cells were treated with drugs for 24 h to determine viability. (*n* = 3 +/– SEM). *p < 0.05 less than corresponding value in siSCR cells. (**D**) TPF-12-293 and TPF-08-196 cells were transfected with an empty vector plasmid (CMV) or with plasmids to express: activated AKT; activated MEK1; activated mTOR; activated STAT3; dominant negative IκB S32A S36A. Twenty-four h after transfection cells were treated with drugs for 24 h to determine viability. (*n* = 3 +/– SEM). *p < 0.05 less than corresponding value in CMV transfected cells.

In addition to BAX, BAK, NOXA and PUMA, knock down of the toxic BH3 domain proteins BIM, BAD or BID also suppressed killing by [pazopanib + AR42] (Figure 4A). Combined drug exposure increased the total expression of BIM and caused BAD S112 dephosphorylation without altering total ERK2 levels (Figure 4B). In Figure 3B we noted that knock down of FADD was more protective than knock down of CD95 at preventing cell killing. Knock down of the death receptors DR4 or DR5 also partially, but significantly, reduced [pazopanib + AR42] killing (Figure 4C). Based on the data showing reduced STAT3, ERK1/2, AKT and mTOR activity, we manipulated cell signaling pathway function. Activation of STAT3, AKT, MEK1 or mTOR suppressed the lethality of [pazopanib + AR42] (Figure 4D). Expression of dominant negative IκB S32A S36A suppressed drug combination toxicity. Thus, based on our collective findings, [pazopanib + AR42] combination lethality proceeds through death receptor signaling (CD95, DR4, DR5); autophagy (AMPK, mTOR, ULK1, ATG13); and ER stress signaling (PERK, eIF2α) that converges on the mitochondrion and that downstream of the mitochondrion cell killing is mediated by AIF and not caspase 9. i.e. we are inducing necroptosis.

**Figure 5:**
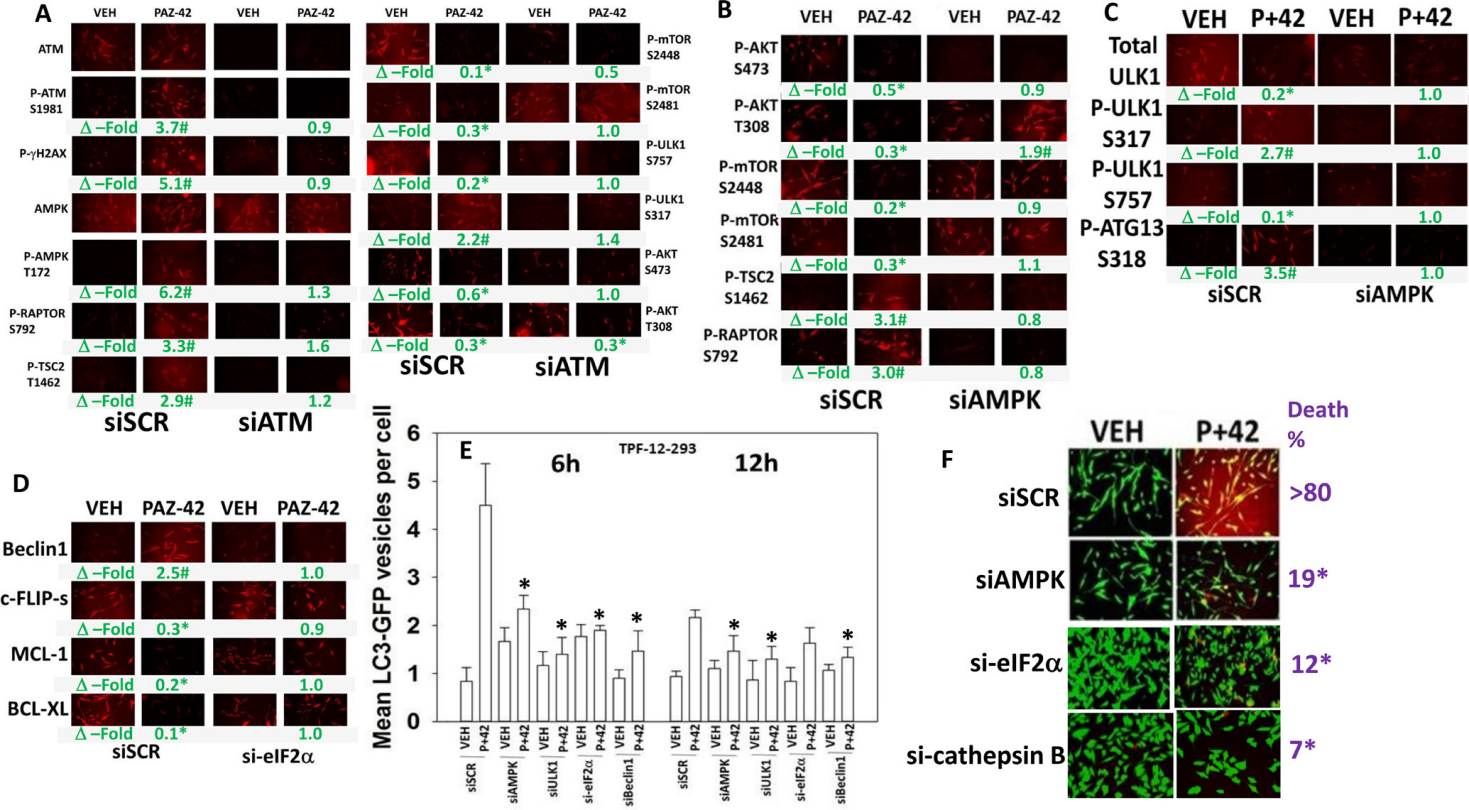
ATM-AMPK signaling and ER stress signaling are required for [pazopanib + AR42] to kill (**A**) TPF-12-293 cells were transfected to knock down the expression of ATM. Twenty-four h after transfection cells were treated with drugs for 6 h. Immuno-fluorescence was performed. (*n* = 3 +/– SEM) ^#^*p* < 0.05 greater than corresponding vehicle control; *p < 0.05 less that corresponding vehicle control. (**B**) and (**C**) TPF-12-293 cells were transfected to knock down the expression of AMPKα. Twenty-four h after transfection cells were treated with drugs for 6 h. Immuno-fluorescence was performed. ^#^*p* < 0.05 greater than corresponding vehicle control; *p < 0.05 less that corresponding vehicle control. (**D**) TPF-12-293 cells were transfected to knock down the expression of eIF2α. Twenty-four h after transfection cells were treated with drugs for 6 h. Immuno-fluorescence was performed. ^#^*p* < 0.05 greater than corresponding vehicle control; *p < 0.05 less that corresponding vehicle control. (**E**) TPF-12-293 cells were transfected with a plasmid to express LC3-GFP and in parallel transfected to knock down expression of AMPKα, ULK1, eIF2α or Beclin1. Twenty-four h after transfection cells were treated with drugs for 6 h or 12 h as indicated. (*n* = 3 +/– SEM). *p < 0.05 less that corresponding value in siSCR cells. (**F**) TPF-12-293 cells were transfected to knock down the expression of AMPKα, cathepsin B or eIF2α. Twenty-four h after transfection cells were treated with drugs for 24 h to determine viability. (*n* = 3 +/– SEM). *p < 0.05 less than corresponding value in siSCR cells.

It has been shown by many groups that HDAC inhibitors can rapidly activate a DNA damage response, i.e. HDAC inhibitors can cause activation of the ataxia telangiectasia mutated protein (ATM) [21, 22]. [Pazopanib + AR42] exposure activated ATM within 6 h that, in agreement with a DNA damage signal, was associated with increased γH2AX phosphorylation (Figure 5A). [Pazopanib + AR42]-induced threonine 172 phosphorylation of the AMPK alpha subunit was ATM-dependent. Drug combination -induced regulatory phosphorylation of RAPTOR and TSC2 required ATM-AMPK signaling (Figure 5A and 5B). Knock down of ATM or of AMPK prevented drug induced phosphorylation of ULK1 S317 and ATG13 S318 and drug-induced dephosphorylation of mTOR S2448, mTOR S2481, AKT T308 and ULK1 S757 (Figure 5A–5C). Thus the data in Figure 3B and Figure 5 argues that DNA damage, through ATM-AMPK signaling, facilitates toxic autophagosome formation. The drug combination activated eIF2α, and knock down of eIF2α prevented the drug-induced declines in MCL-1, BCL-XL and c-FLIP-s expression and the increase in Beclin1 expression (Figure 5D). Knock down of AMPK, ULK1, Beclin1 or eIF2α suppressed the increase in toxic autophagosome levels (Figure 5E). Knock down of AMPK, eIF2α or the lysosomal protease cathepsin B reduced drug-induced cell killing (Figure 5F). Thus, ER stress signaling, via eIF2α and Beclin1, facilitates toxic autophagosome/autolysosome formation and cathepsin B activation, and eIF2α signaling, via reduction of c-FLIP-s, MCL-1 and BCL-XL levels, facilitates death receptor signaling and mitochondrial dysfunction.

**Figure 6:**
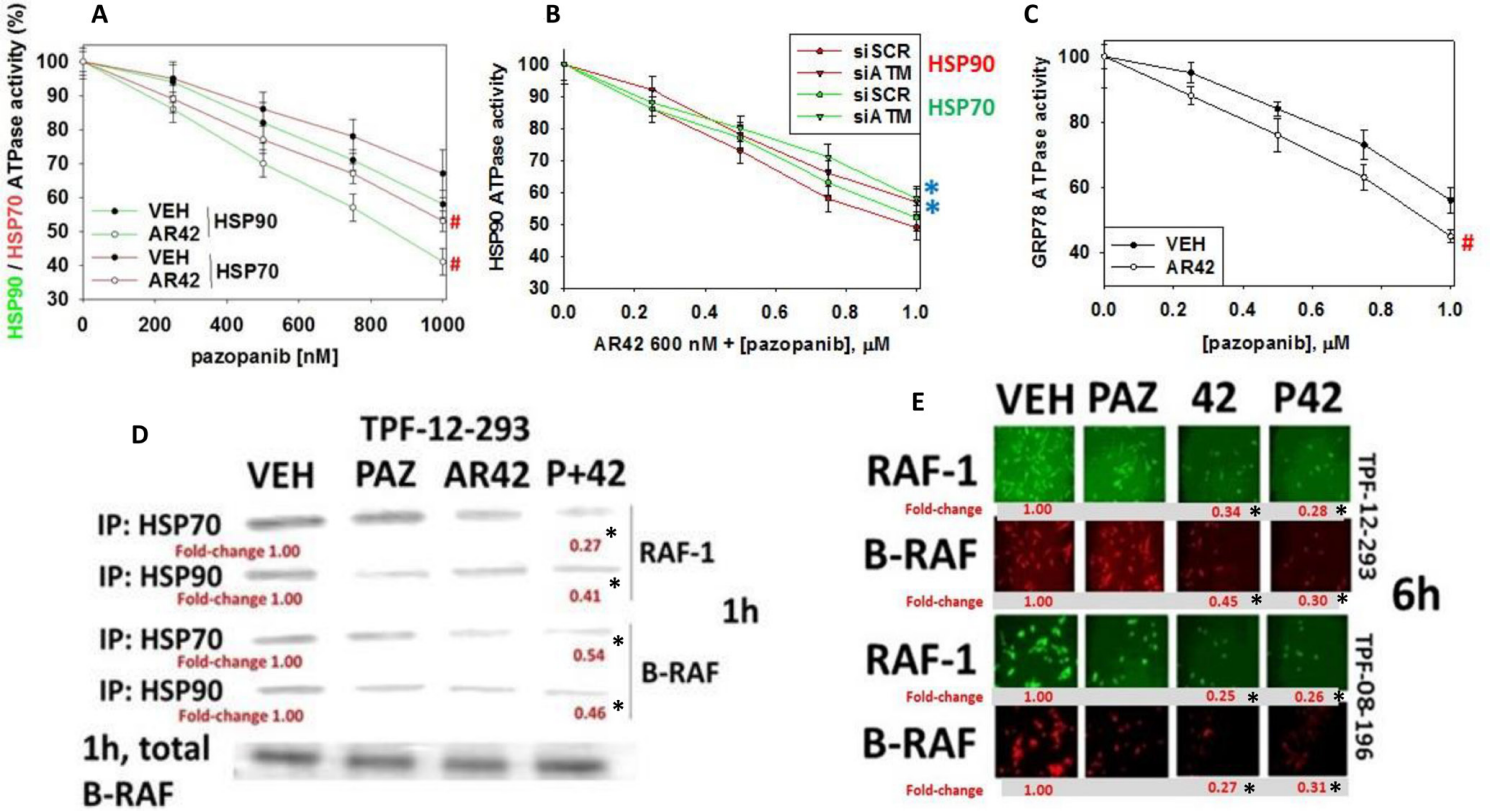
[Pazopanib + AR42] regulates chaperone function (**A**) TPF-12-293 cells were treated with drugs and the dose-response of chaperone ATPase activity in response to increasing concentrations of pazopanib *in vitro* is plotted (*n* = 3 +/– SEM) ^#^*p* < 0.05 greater inhibition than vehicle control. (**B**) TPF-12-293 cells were transfected to knock down ATM expression. Twenty-four h after transfection cells were treated with drugs and the dose-response of chaperone ATPase activity in response to increasing concentrations of pazopanib *in vitro* is plotted (*n* = 3 +/– SEM) *p < 0.05 less inhibition than siSCR control. (**C**) TPF-12-293 cells were treated drugs and the dose-response of chaperone ATPase activity in response to increasing concentrations of pazopanib *in vitro* is plotted (*n* = 3 +/– SEM) ^#^*p* < 0.05 greater inhibition than vehicle control. (**D**) and (**E**) TPF-12-293 cells were either not transfected or transfected with plasmids to express either FLAG-HSP90 or HA-HSP70. Twenty-four h after transfection cells were treated with drugs as indicated. For transfected cells after 1 h, HSP90 and HSP70 were immunoprecipitated and the amount of RAF-1 and B-RAF co-precipitating with the chaperones determined by SDS PAGE and western blotting. For non-transfected TPF-12-293 and TPF-08-196 cells after 6 h, cells were fixed in place and immuno-fluorescence performed to detect the total expression of RAF-1 and of B-RAF (*n* = 3 +/– SEM) *p < 0.05 less than vehicle control value.

It has been known for several decades that both HSP90 and HSP70 chaperone B-RAF and RAF-1 and that removal of these chaperones during ‘RAF’ chromatographic protein purification results in rapid RAF protein denaturation [23–26]. Prior studies have demonstrated that pazopanib is an inhibitor of the chaperones HSP90 and HSP70 that was associated with a tertiary structural change in the NH2-termini of the proteins [7, 11]. Using vemurafenib resistant TPF-12-293 cells we re-confirmed that pazopanib could alter the structure of chaperones in melanoma cells as judged by reduced immuno-fluorescence at the NH2-termini but not at their COOH termini (Supplementary Figure 5). Notably for HSP90 and HSP70, AR42 treatment of the cells enhanced the ability of pazopanib to occlude the epitope at the NH2-termini of the chaperone proteins. In agreement with this observation, although AR42 treatment did not alter basal chaperone activities, *in vivo* treatment of chaperones with the HDAC inhibitor facilitated the ability of pazopanib to inhibit the HSP90 and HSP70 ATPase activities *ex vivo* (Figure 6A). Of greater interest, the ability of AR42 to interact with pazopanib to suppress chaperone ATPase activity was prevented by knock down of ATM (Figure 6B). Pazopanib was also shown to be an inhibitor of the GRP78 ATPase activity, an effect that was enhanced by prior exposure of cells to AR42 (Figure 6C). Treatment of melanoma cells with [pazopanib + AR42] for one hour significantly reduced the amount of B-RAF and RAF-1 that co-precipitated with HSP90 and HSP70 (Figure 6D). Furthermore, in agreement with reduced ‘RAF’ protein-chaperone association and greater ‘RAF’ denaturation, treatment of the melanoma cells for 6 h with AR42 or with [pazopanib + AR42] reduced the total protein levels of B-RAF and RAF-1 (Figure 6E).

**Figure 7:**
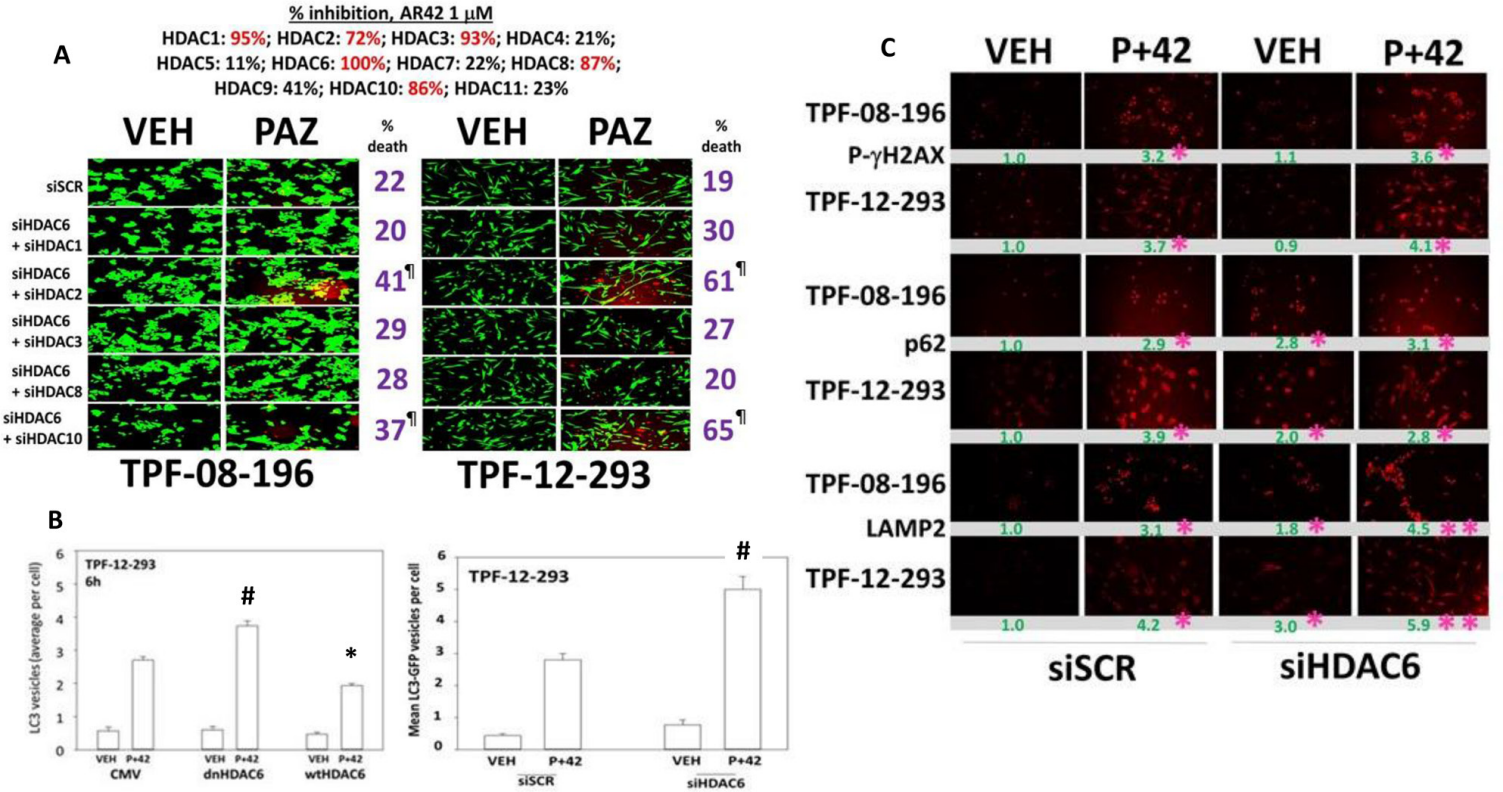
HDAC6 regulates autophagosome formation (**A**) Upper: The percentage catalytic inhibition of each HDAC by AR42 at a concentration of 1 μM; Lower: Melanoma cells were transfected to knock down the expression of HDAC6, combined with molecules to knock down the expression of HDAC1 or HDAC2 or HDAC3 or HDAC8 or HDAC10. Twenty-four h after transfection the cells were treated with pazopanib to determine viability (*n* = 3 +/– SEM). ^¶^*p* < 0.001 greater than corresponding values in siSCR; [siHDAC6 + siHDAC1]; [siHDAC6 + siHDAC3]; [siHDAC6 + siHDAC8] cells. (**B**) Melanoma cells were transfected with: left graph: an empty vector plasmid, a plasmid to express wild type HDAC6 or a plasmid to express dominant negative HDAC6; right graph: to knock down expression of HDAC6. In parallel all cells were transfected with a plasmid to express LC3-GFP. Twenty-four h after transfection cells were treated with drugs for 6 h. The numbers of punctate LC3-GFP staining vesicles were counted (*n* = 3 +/– SEM) ^#^*p* < 0.05 greater than corresponding value in siSCR/CMV cells; *p < 0.05 less than corresponding value in siSCR/CMV cells. (**C**) Melanoma cells were transfected to knock down expression of HDAC6. Twenty-four h after transfection cells were treated with drugs for 6 h. Staining was performed to determine the levels of P-γH2AX; p62/SQSMT1; LAMP2. (*n* = 3 +/– SEM) *p < 0.05 greater than vehicle control in siSCR cells; **p < 0.05 greater than corresponding value in siSCR cells.

AR42 is known to be a highly efficacious catalytic inhibitor of HDAC1, HDAC2, HDAC3, HDAC6, HDAC8 and HDAC10 (Figure 7A, upper). Individual knock down of HDAC1/2/3/6/8/10 did not significantly alter basal levels of cell viability and only knock down of HDAC6 modestly enhanced pazopanib lethality (data not shown). Knock down of HDAC6 combined with knock down of HDAC2 or of HDAC10 caused a significant high level of killing when combined with pazopanib (Figure 7A, lower). The HSP90-associated HDAC, HDAC6, has been proposed to regulate autophagic flux, with protein acetylation reducing the maturation of autophagosomes into autolysosomes [27–30]. To test for a role of HDAC6 in our system, we over-expressed HDAC6, either using a catalytically active wild type protein or a protein that lacks activity in the two deacetylase catalytic activity sites. In TPF-12-293 cells the absence of HDAC6 expression or in the presence of dominant negative HDAC6, drug combination exposure caused a significantly higher induction of LC3-GFP punctae in cells (Figure 7B). Over-expression of wild type HDAC6 reduced the induction of GFP+ punctae. Similar data were obtained in TPF-08-196 cells (Supplementary Figure 6). In the absence of HDAC6 expression the basal levels of p62 and LAMP2 were also elevated; in the case of p62 the drug combination did not further enhance its levels; in the case of LAMP2 the drug combination did further increase its levels (Figure 7C). Knock down of HDAC6 did not alter the ability of the drug combination to elevate P-γH2AX levels arguing that the drug combination effects were more likely proximal to the autophagic apparatus than to ATM and DNA damage.

**Figure 8:**
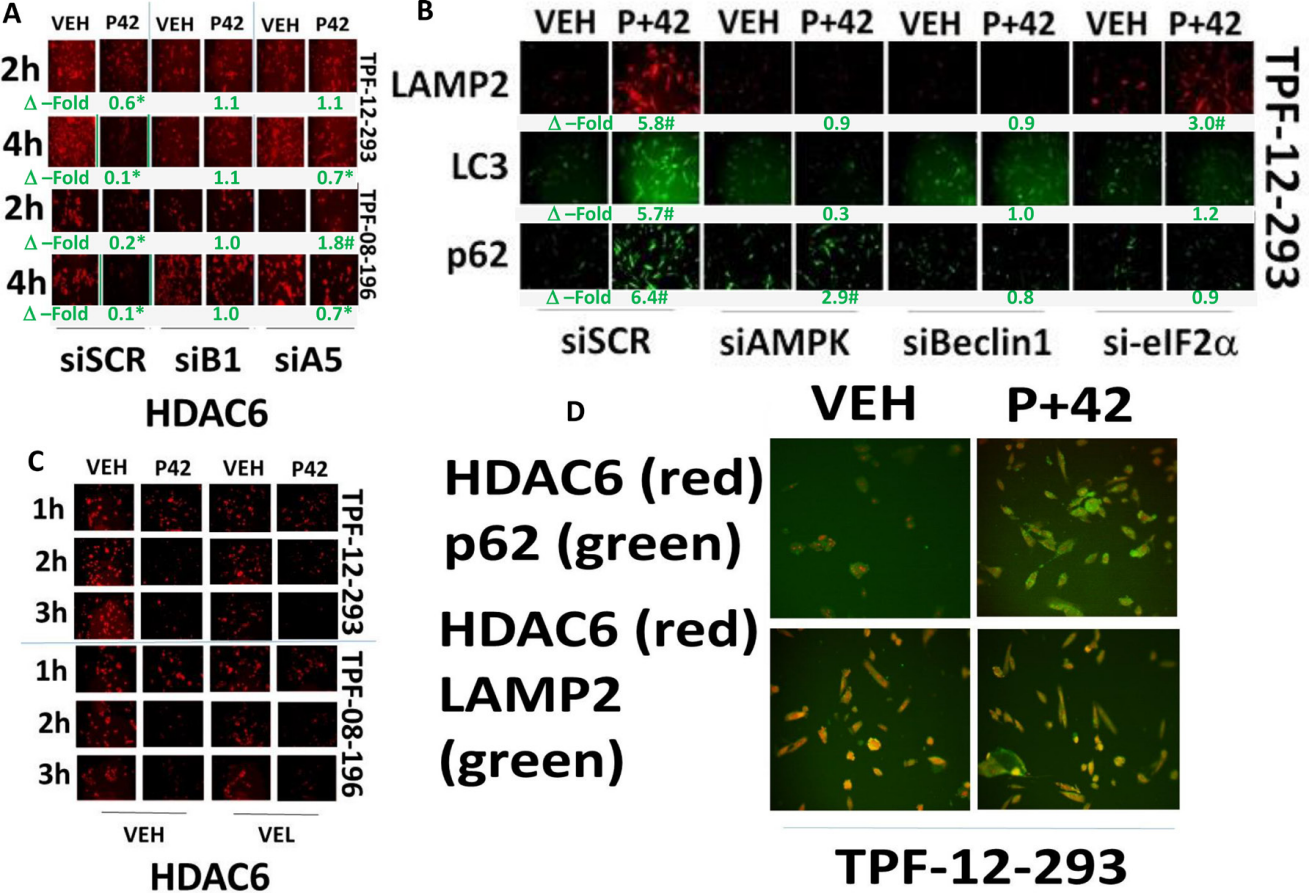
AR42 down-regulates HDAC6 expression through AMPK/eIF2α-autophagy signaling (**A**) TPF-12-293 and TPF-08-196 cells were transfected to knock down the expression of Beclin1 (B1) or ATG5 (A5). Twenty-four h after transfection cells were treated for 6 h with drugs and the expression of HDAC6 was determined (*n* = 3 +/– SEM) *p < 0.05 lower staining intensity than corresponding intensity in vehicle control treated cells; ^#^*p* < 0.05 greater staining intensity than corresponding intensity in vehicle control treated cells. (**B**) Melanoma cells were transfected to knock down expression of AMPKα, eIF2α or Beclin1. Twenty-four h after transfection cells were treated with drugs for 6 h and the expression of LAMP2, LC3 and p62/SQSMT1 determined (*n* = 3 +/– SEM) *p < 0.05 lower staining intensity than corresponding intensity in vehicle control treated cells; ^#^*p* < 0.05 greater staining intensity than corresponding intensity in vehicle control treated cells. (**C**) TPF-12-293 and TPF-08-196 cells were treated for 6 h with drugs in the presence or absence of Velcade (10 nM). The expression of HDAC6 was determined. There is no statistically significant difference in the reduction of HDAC6 regardless of the presence of bortezomib. (**D**) Melanoma cells were treated with drugs for 6 h and the co-localization of HDAC6 with p62/SQSMT1 and with LAMP2 determined.

During these studies examining HDACs, we surprisingly discovered that AR42 also rapidly reduced the *protein* levels of HDAC2, HDAC5, HDAC6, HDAC10 and HDAC11 (Supplementary Figure 7A and 7B). Similar data showing reduced HDAC levels were obtained using valproate (Supplementary Figure 8A). Melanoma cells, compared to other tumor cell types, expressed higher protein levels of HDAC6 which may explain melanoma sensitivity to HDAC inhibitors (Supplementary Figure 8B). We next attempted to define the mechanisms by which HDAC6 protein expression was being rapidly reduced. Knock down of ATG5, an early essential inducer of autophagosome formation, or knock down of Beclin1, an intermediate essential inducer of autophagosome formation, both prevented [pazopanib + AR42] treatment from reducing HDAC6 expression (Figure 8A). Thus, HDAC6 is degraded through a process involving “autophagy.” Treatment of melanoma cells with [pazopanib + AR42] after 6 h increased the protein levels of LAMP2, LC3 and p62/SQSTM1 (Figure 8B). This effect was reduced by knock down of AMPK, Beclin1 and to a lesser extent by eIF2α. In contrast to a regulatory role for autophagy in the levels of HDAC6 expression, the clinically relevant inhibitor of E3 ligase activities, bortezomib (Velcade^®^), did not modify the reduction in HDAC6 expression (Figure 8C). In cells treated with [pazopanib + AR42], at 10× magnification HDAC6 co-localized with LAMP2, but not p62/SQSTM1, validating a lysosomal -dependent degradation mechanism (Figure 8D).

As stated previously, HSP90 is an acetylated protein, and HDAC6 has been proposed to de-acetylate HSP90. Acetylation of HSP90 has been proposed to reduce the chaperoning ability of HSP90. Knock down of HDAC6 elevated the basal levels of HSP90 acetylation and prevented [pazopanib + AR42] from increasing HSP90 acetylation (Supplementary Figure 9A). Treatment of cells with [pazopanib + AR42] significantly enhanced HSP90 acetylation which correlated with reduced HDAC6 expression (Supplementary Figure 9B). Knock down of AMPK, which prevents both autophagosome/autolysosome formation and cell killing, prevented the increase in HSP90 acetylation and prevented the down-regulation of HDAC6 (Supplementary Figure 9C). At 60× magnification, [pazopanib + AR42] exposure increased the levels of staining for LAMP2 and for ATG13 S318 that both appeared to be contained in small vesicles (Supplementary Figure 9D). However, LAMP2 and P-ATG13 S318 did not co-localize. Together with the data in Figure 8D, this argues that HDAC6 after combined drug exposure is localized in autolysosomes.

**Figure 9:**
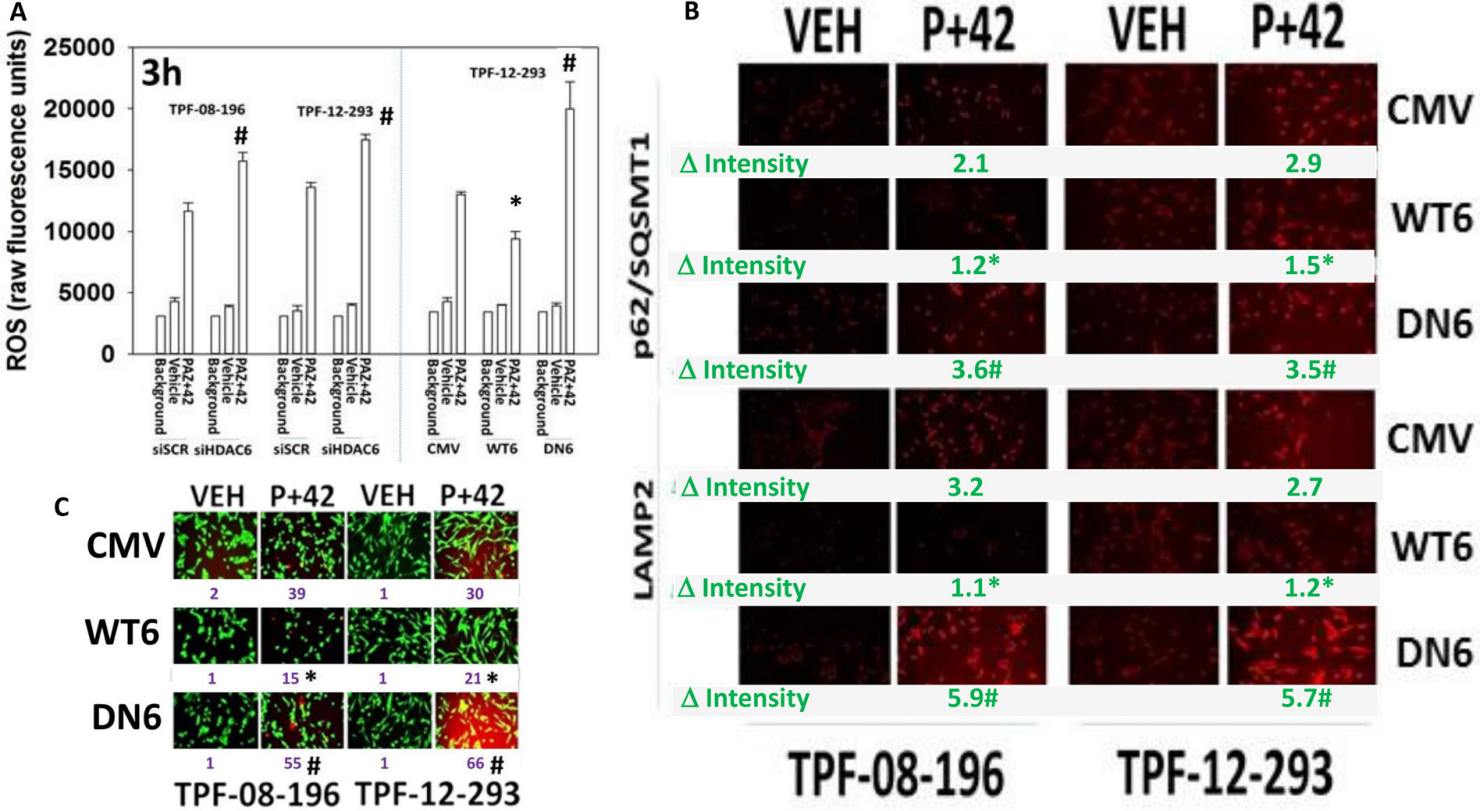
HDAC6 expression regulates the production of reactive oxygen species and tumor cell killing (**A**) Melanoma cells were transfected to knock down expression of HDAC6. In parallel they were transfected as indicated with an empty vector plasmid (CMV) or with plasmids to express active wild type HDAC6 (WT6) or a catalytically inactive HDAC6 (DN6). Twenty-four h after transfection cells were treated with drugs for 3 h. The level of reactive oxygen species (ROS) was determined (*n* = 3 +/– SEM) ^#^*p* < 0.05 greater than corresponding value in siSCR/CMV cells; *p < 0.05 less than corresponding value in CMV cells. (**B**) Melanoma cells were transfected with an empty vector control plasmid (CMV), or with plasmids to express wild type HDAC6 or dominant negative HDAC6. Twenty-four h after transfection, cells were treated with drugs for 6 h and the expression of p62/SQSMT1 and LAMP2 determined. (**C**) Melanoma cells were transfected with an empty vector control plasmid (CMV), or with plasmids to express wild type HDAC6 or dominant negative HDAC6. Twenty-four h after transfection, cells were treated with drugs for 24 h to determine viability (*n* = 3 +/– SEM). ^#^*p* < 0.05 greater than corresponding value in CMV cells; *p < 0.05 less than corresponding value in CMV cells.

Dysregulation of HDAC6 has also been linked to elevated production of reactive oxygen species and our prior data had shown the drug combination reduced TRX and SOD2 levels. Knock down of HDAC6 enhanced the abilities of our drug combination treatment to increase ROS levels (Figure 9A). Over-expression of TRX or SOD2 protected cells from drug combination lethality (data not shown). Over-expression of HDAC6 reduced the ability of [pazopanib + AR42] to increase ROS levels. Expression of dominant negative HDAC6 enhanced the ability of [pazopanib + AR42] to increase ROS levels. Knock down of HDAC6 was previously shown to enhance the ability of [pazopanib + AR42] to increase LAMP2 levels, and similarly, expression of dominant negative HDAC6 enhanced drug-induced LAMP2 levels (Figure 9B). Expression of wild type HDAC6 lowered the basal levels of LAMP2 and significantly reduced the accumulation of LAMP2 after drug exposure. Similar confirmatory data were also obtained when we examined changes in p62/SQSMT1 expression. The alterations observed in autophagosome formation were also reflected in the ability of [pazopanib + AR42] to kill the melanoma cells, with expression of wild type HDAC6 reducing killing and expression of dominant negative HDAC6 promoting death (Figure 9C).

**Figure 10:**
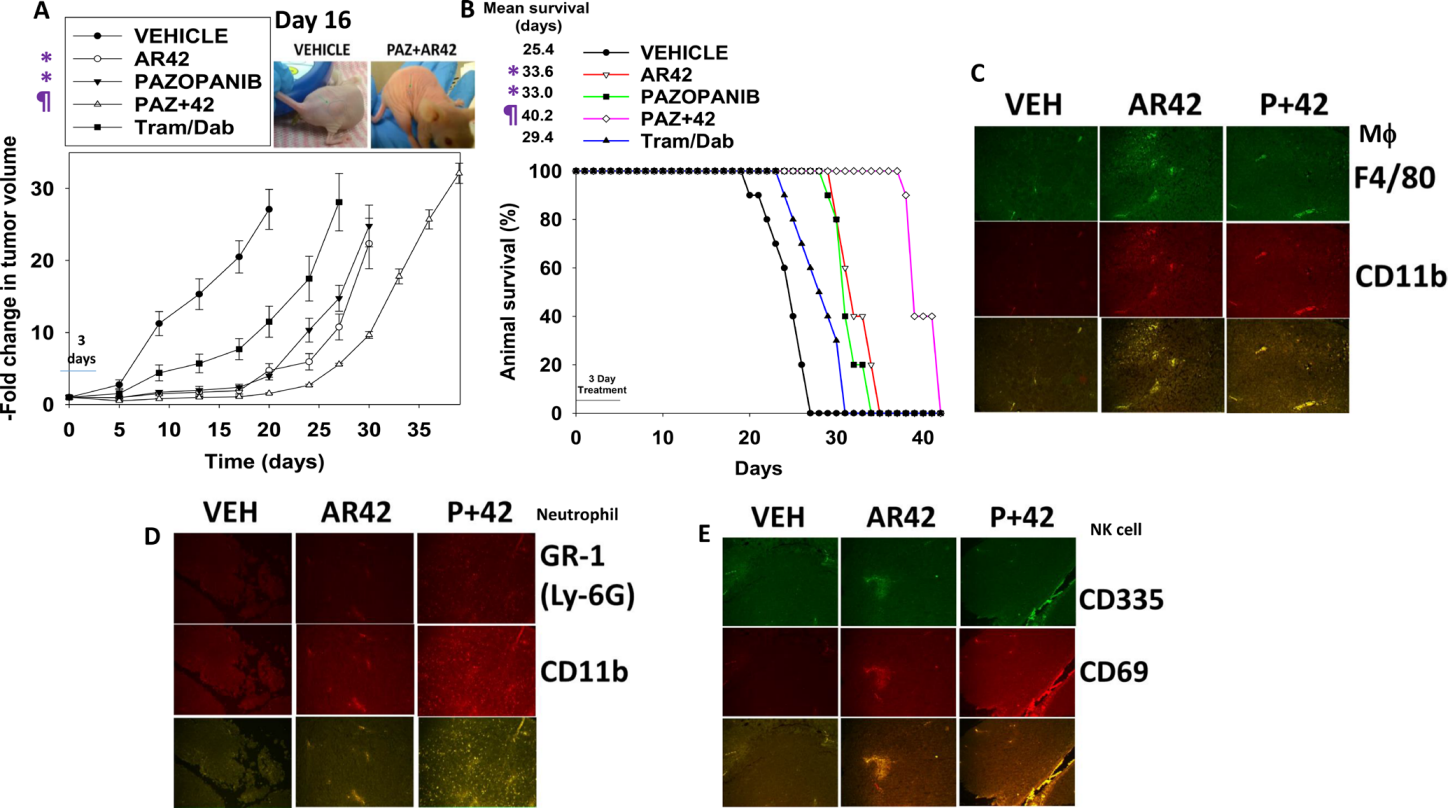
[Pazopanib + AR42] suppresses melanoma tumor growth *in vivo* (**A**) Tumor volumes were measured at the start of treatment (Day 0) and on the indicated days (*n* = 10, +/– SEM). *p < 0.05 less than Trametinib/Dabrafenib treated tumors; ^¶^*p* < 0.05 less than AR42 alone or pazopanib alone treated tumors. (**B**) Animals were sacrificed when the tumor volume reached 250 mm^3^. Survival data are plotted as a Kaplan Meier survival curve. *p < 0.05 greater survival compared to vehicle control; ^¶^*p* < 0.05 greater survival compared to AR42 alone. (**C**–**E**) Four micron slides of vehicle treated; AR42 treated; and [pazopanib + AR42] tumors were made and immunohistochemistry performed to determine the expression and co-localization of: C. F4/80 and CD11b (macrophages); D. GR-1 and CD11b. Note that GR-1 staining used a conjugated antibody that was detected in the Far Red range. The hue of the image was converted to green to permit co-localization analyses with CD11b (neutrophil); E. CD335 and CD69 (natural killer cell).

We next performed animal studies to validate the *in vitro* findings. Initial studies used MEL28 cells made resistant to trametinib/dabrafenib by prolonged culture in 2 μM trametinib/2 μM dabrafenib. One million tumor cells were implanted into the rear flanks of athymic mice and seven days later when the mean tumor volume was 9.0 +/– 0.8 mm^3^, animals were treated with drugs for three days. Vehicle control treated tumors grew rapidly and within 20 days of implantation tumor volumes had begun to reach the sacrifice cut-off point of 250 mm^3^ (the equivalent of a 0.75 kg tumor in a 60 kg human) (Figure 10A and 10B). The mean time to death for vehicle treated animals was 25.4 +/– 0.7 days. Trametinib/dabrafenib treated tumors grew more slowly than control tumors with a mean animal survival of 29.4 +/– 1.0 days, which however, when also taking into account the three-day growth-arresting drug exposure was not a statistically different survival value from the control animal group. Animals exposed to either AR42 or pazopanib had mean survival values of 33.6 +/– 0.7 days and 33.0 +/– 0.6 days, respectively. These increases in animal survival were significant (*p* < 0.01). Animals exposed to pazopanib AR42 had a mean survival of 40.2 +/– 0.6 days which was significantly greater than the survival of animals treated only with AR42 or with pazopanib (*p <* 0.02). Thus, a combined three day pazopanib plus two day AR42 treatment using clinically proportional drug doses had increased animal survival from 25.4 days to 40.2 days (∼14–15 days), a 58% increase.

Using tumors isolated at the time of sacrifice, 25–40 days after drug exposure, we obtained 4 micron sections and performed immuno-histochemistry analyses to determine the expression of biomarkers within the tumor (Figure 10C–10E). When compared to vehicle control treated tumors, tumors previously exposed to AR42 or to [pazopanib + AR42] exhibited higher levels of: macrophages; neutrophils; and natural killer cells (Figure 10C–10E). Additional blown up images of the data in Figure 10C–10E are provided in Supplementary Figures 10–19. After examining multiple tumor sections from multiple tumors, it became evident that drug exposure, particularly exposure to AR42 as a single agent, had dramatically altered the cyto-architecture of the tumors (Supplementary Figures 20–23). Prior AR42 treatment had caused the re-grown tumors to become less dense with interstitial spaces between defined “clumps” of tumor cells.

**Figure 11:**
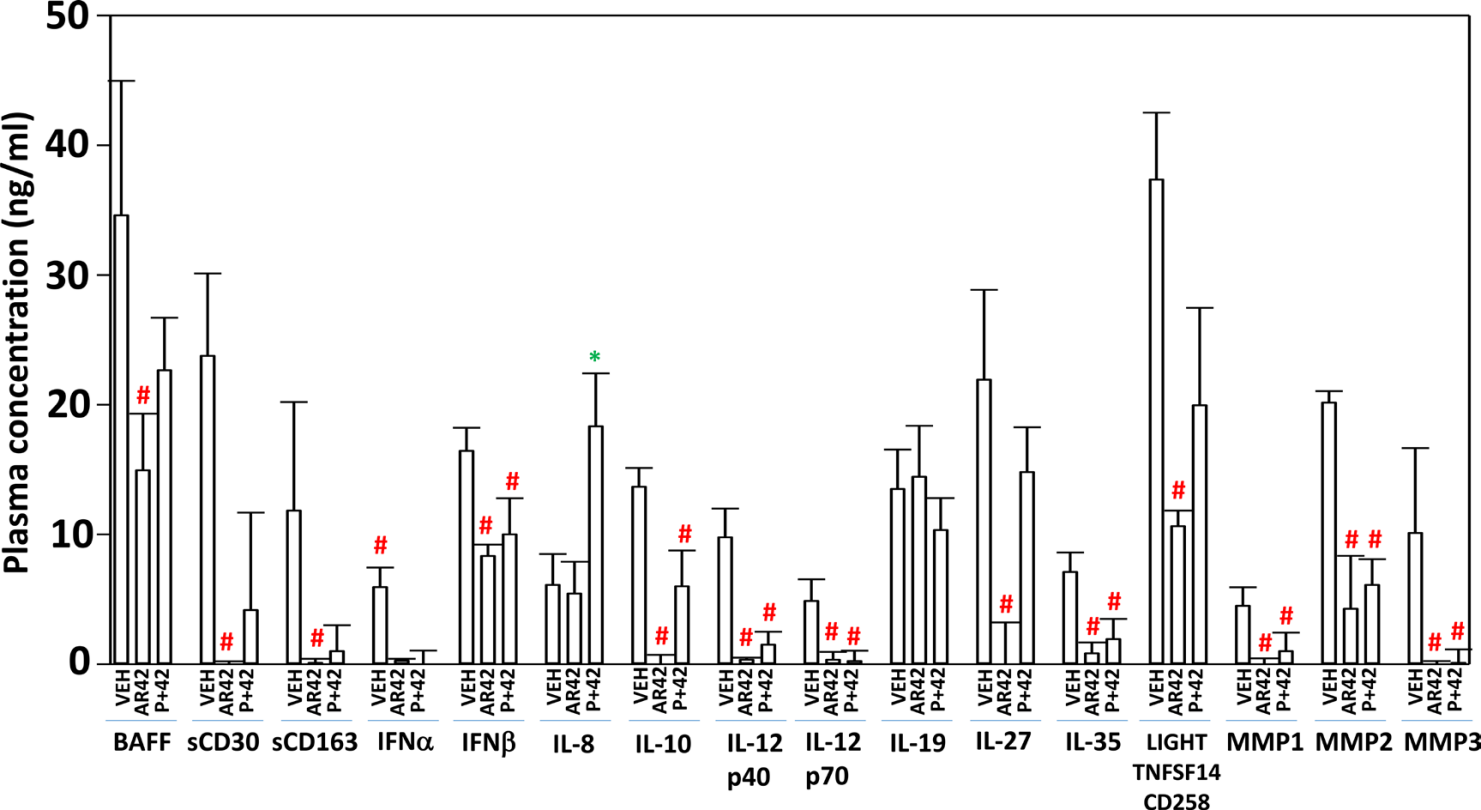
AR42 and [pazopanib + AR42] reduce plasma levels of MMP1, MMP2, MMP3 and IL-10 and increase plasma IL-8 levels As tumor volumes reached 250 mm^3^, animals were sacrificed and blood and tumor obtained. Clarified plasma and tumor cell lysates were then subjected to multiplex assays as described in the Methods to detect the plasma levels of the indicated cytokines and phosphorylation status of signal transduction proteins using a Bio-Rad MAGPIX multiplex instrument (total 3 animals per condition, +/– SEM). ^#^*p* < 0.05 less than corresponding value from vehicle control treated animals.

**Figure 12:**
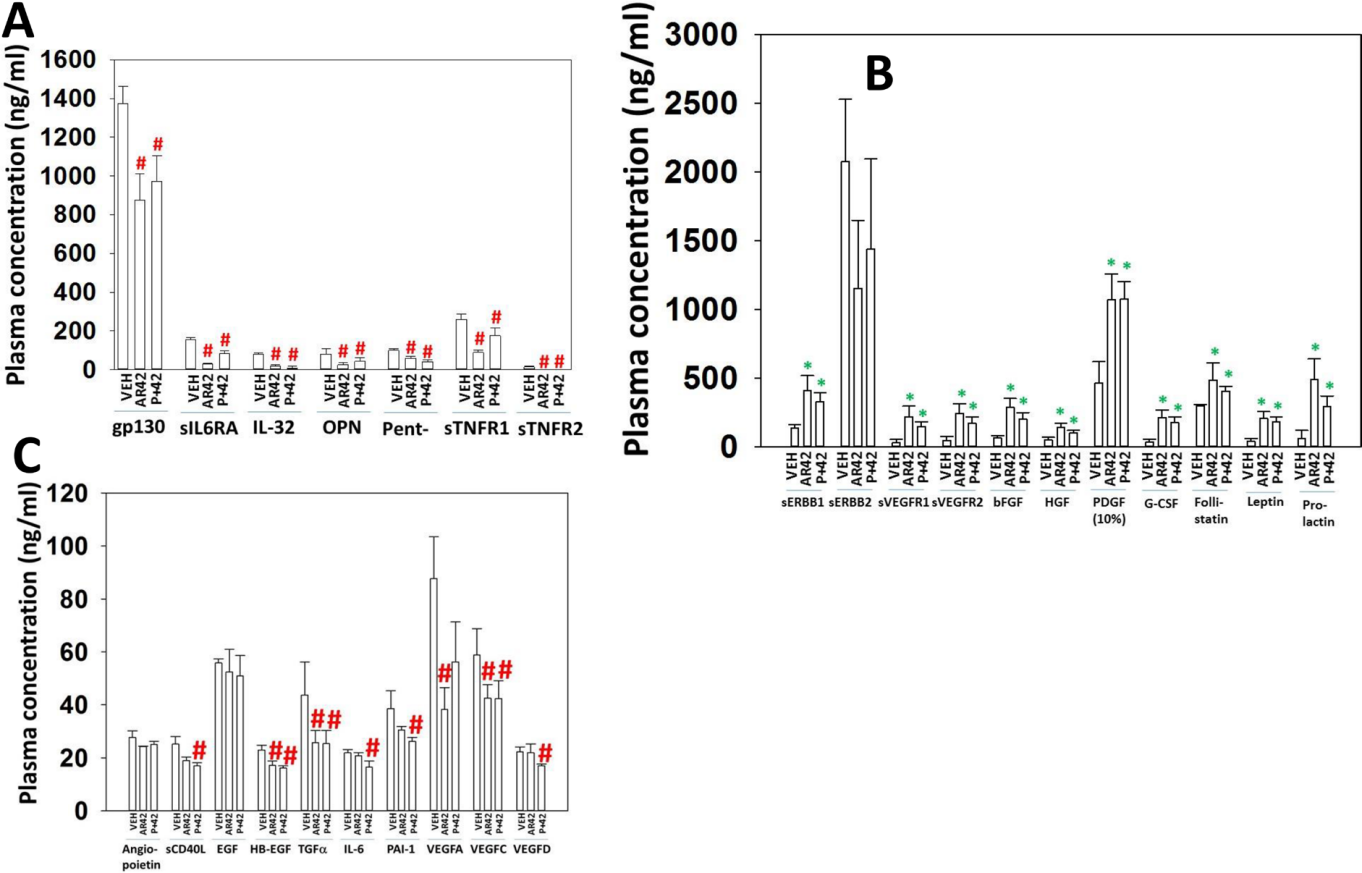
AR42 and [pazopanib + AR42] induce compensatory survival through expression of PDGF, bFGF, HGF and prolactin (**A**–**C**) As tumor volumes reached 250 mm^3^, animals were sacrificed and blood and tumor obtained. Clarified plasma and tumor cell lysates were then subjected to multiplex assays as described in the Methods to detect the plasma levels of the indicated cytokines and phosphorylation status of signal transduction proteins using a Bio-Rad MAGPIX multiplex instrument (total 3 animals per condition, +/– SEM). ^#^*p* < 0.05 less than corresponding value from vehicle control treated animals; *p < 0.05 greater than corresponding value from vehicle control treated animals.

Using tumor material and mouse plasma isolated at the time of animal sacrifice, approximately 25–40 days after cessation of drug exposure, we next performed antibody array multiplex analyses on tissue and plasma to detect changes in protein phosphorylation and altered mouse plasma levels of *human* growth factors and cytokines. Plasma from animals exposed to AR42, and generally though to a lesser extent [pazopanib + AR42], had reduced levels of human soluble CD163, human interferon alpha, interferon beta, interferon gamma (not shown), IL-10, IL-12 family cytokines and the metalloproteases MMP1-3 (Figure 11). That less sCD163 in the plasma was detected after the drug treatments implies that macrophage activity had declined; future studies will need to define the M1/M2 macrophage phenotype found within the tumors. MMP1, MMP2 and MMP3 are known to be essential proteases for both the growth and metastatic spread of melanoma tumors [37]. In animals exposed to [pazopanib + AR42] the plasma levels of IL-8 were increased, a finding similar to our prior studies using [pemetrexed + sorafenib] and [ruxolitinib + afatinib] [38, 39]. Both the AR42 and [pazopanib + AR42] treatments selected for lower plasma levels of tumor-derived human gp130 and of soluble IL-6RA (Figure 12A). IL-6 in the plasma binds to soluble IL-6RA and this complex has elevated agonist activity at activating tumor cell surface IL-6RA, however, gp130 in the plasma binds to the IL-6/sIL-6RA complex and quenches the ability of the protein complex to act as an agonist [40]. The normal plasma concentration range in a healthy human for gp130 is 200–400 ng/ml and that of IL-6RA is 30–70 ng/ml. Our data demonstrated that AR42 reduced gp130 levels by ∼40% and those of IL-6RA by ∼80% which collectively argues that overall IL-6 paracrine/autocrine signaling may be reduced in AR42 treated melanoma tumors.

In contrast to our data examining the reduced expression of cytokines, both AR42 and [pazopanib + AR42] exposure increased the plasma levels of many growth factors including bFGF, HGF, PDGF, G-CSF and prolactin, though not of IL-6 which further validates our initial hypothesis over IL-6 signaling (Figure 12B and 12C). The basal plasma levels of PDGF were approximately 100-fold greater than those of the other growth factors examined in our panel, and, comparing the ordinate scale of panel B to the scaling in panel A and panel C, we conclude that transient AR42 exposure causes the further evolution of MEL28-R melanoma cells that are very likely becoming addicted to the signaling downstream of the PDGFRα/PDGFRβ/FGFR/c-MET/prolactin receptors. In a manner reminiscent of IL-6, the functionality of signaling via ERBB1 was also likely reduced following drug exposure as both the levels of HB-EGF and TGFα declined in drug treated plasma and the levels of soluble ERBB1, that will sequester and inactivate circulating HB-EGF and TGFα increased.

**Figure 13:**
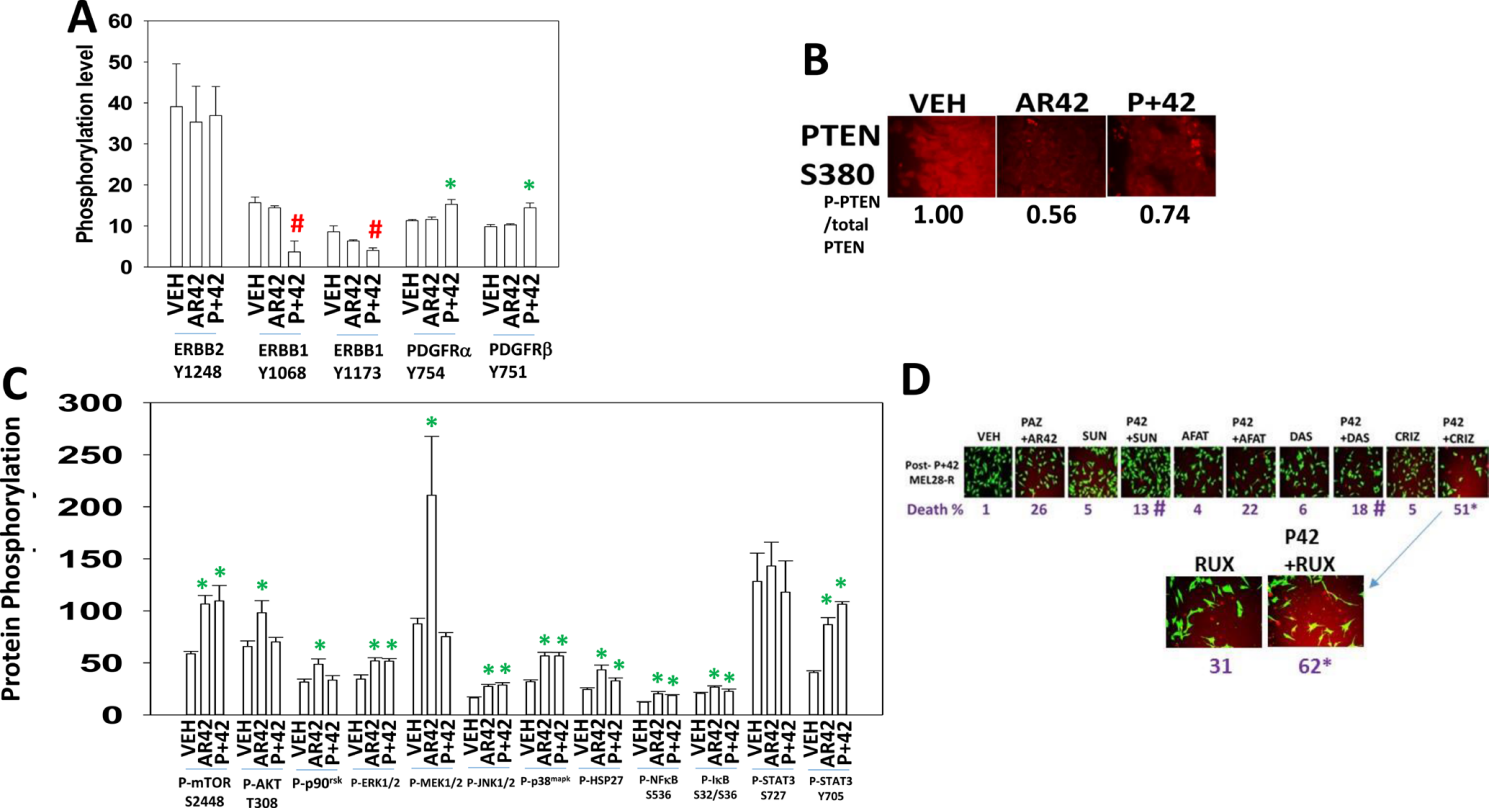
AR42 and [pazopanib + AR42] reduce signaling by ERBB1 and induce signaling through the PDGFR (**A**–**C**) As tumor volumes reached 250 mm^3^, animals were sacrificed and blood and tumor obtained. Clarified plasma and tumor cell lysates were then subjected to multiplex assays as described in the Methods to detect the plasma levels of the indicated cytokines and phosphorylation status of signal transduction proteins using a Bio-Rad MAGPIX multiplex instrument (total 3 animals per condition, +/– SEM). ^#^*p* < 0.05 less than corresponding value from vehicle control treated animals; *p < 0.05 greater than corresponding value from vehicle control treated animals. (**D**) MEL28-R tumor cells were isolated from tumors previously treated with [pazopanib + AR42]. Cells were treated with drugs in the presence or absence of: afatinib (0.5 μM); sunitinib (0.5 μM); dasatinib (0.5 μM); ruxolitinib (0.5 μM); or crizotinib (0.5 μM). Tumor cells were isolated 12 h after treatment and cell viability determined (*n* = 3 +/– SEM) *p < 0.05 greater than corresponding value in [pazopanib + AR42] cells treated with vehicle control; ^#^*p* < 0.05 less than corresponding value in [pazopanib + AR42] cells treated with vehicle control.

We then examined the phosphorylation and expression of signal transduction regulatory proteins in the tumors themselves. In agreement with reduced HB-EGF and TGFα levels and increased soluble ERBB1, the activity of ERBB1 was reduced in both AR42 and [pazopanib + AR42] treated tumors (Figure 13A). In contrast, the activity of PDGFRα and PDGFRβ were both elevated after [pazopanib + AR42] exposure which correlates with elevated plasma levels of PDGF. Downstream of growth factor receptors the expression level of the lipid phosphatase PTEN was reduced as was the inhibitory phosphorylation at PTEN S380 (Figure 13B). As the phosphorylation of PTEN was reduced to a greater extent than its total protein level, our data argue that PTEN activity, despite the reduction in total PTEN expression, may be elevated in the drug exposed tumors. *Nota bene*: in general phosphatases are known to be approximately 10-fold more catalytically active than kinases, thus a small change in phosphatase activity will be magnified on the regulation of its substrate. Downstream of PTEN we discovered that AR42 had activated AKT ∼1.7-fold whereas in [pazopanib + AR42] treated tumors no AKT activation was apparent (Figure 13C). Also downstream of PTEN, the phosphorylation of mTOR S2448 was enhanced in both AR42 and [pazopanib + AR42] treated tumors. The MEL28-R cells initially implanted into the mice were dabrafenib/trametinib resistant and in drug exposed tumors our data argued that MEK1/2-ERK1/2-p90^rsk^ signaling was uncoupled. Prior [pazopanib + AR42] exposure had no effect on p90^rsk^ phosphorylation or on MEK1/2 phosphorylation whereas it enhanced ERK1/2 phosphorylation. The prior drug treatments, in a manner similar to that observed for mTOR and ERK1/2, modestly enhanced the phosphorylation of JNK1/2, p38^MAPK^ and the substrate of p38^MAPK^, HSP27 by ∼1.7-fold. The phosphorylation of STAT3 at S727 was not altered by drug exposure whereas the phosphorylation of STAT3 Y705 was elevated ∼2.6-fold in drug combination treated cells. The phosphorylation of NFκB S536 was enhanced ∼1.5-fold in drug treated tumors and the phosphorylation of IκB S32 S36 enhanced ∼1.1-fold in drug treated cells, together arguing for modestly enhanced NFκB activity in drug treated cells.

Finally, as a standard of care drug in sarcoma and renal carcinoma pazopanib is dosed daily and would thus be likely to block any compensatory survival signaling through PDGFRα/PDGFRβ/FGFR, but not through c-MET or SRC-linked cytokine receptors. Hence, we determined in MEL28-R cells isolated from tumors previously treated with [pazopanib + AR42] whether inhibition of c-MET could enhance cell killing. Post- [pazopanib + AR42] MEL28-R cells were still sensitive to being rapidly (12 h) killed by [pazopanib + AR42] (Figure 13D). Neither sunitinb (an inhibitor of G-CSFR, FLT3, KIT, PDGFR, VEGFR), dasatinib (an inhibitor of BCR-ABL, SRC family, KIT, Ephrin-Rs) nor afatinib (an inhibitor of ERBB1, ERBB2, ERBB4) enhanced the lethality of [pazopanib + AR42]. In contrast, the relatively specific c-MET and ALK inhibitor crizotinib significantly enhanced [pazopanib + AR42] lethality. Downstream of c-MET, the JAK1 and JAK2 specific inhibitor ruxolitinib also enhanced [pazopanib + AR42] lethality. Thus, our data, derived from multiplex assays and the observation of PDGF over-expression and STAT3 activation in evolved tumors, validates the concept of using crizotinib or ruxolitinib as a third agent in combination with [pazopanib + AR42] to enhance tumor cell killing and to circumvent the development of drug resistance.

## DISCUSSION

The present studies were initially conducted to determine whether the drug combination of [pazopanib + AR42], previously shown to kill sarcoma and renal carcinoma cells and now the subject of a phase I trial, could also kill melanoma cells expressing a mutated active B-RAF protein. In the trial NCT02795819, 3 out of 4 renal carcinoma patients in the lowest dosing cohort have exhibited one PR and two prolonged SDs (Poklepovic and Dent, unpublished observations). Using multiple PDX models of mutant B-RAF melanoma we determined that AR42 *in vitro* was a more potent anti-melanoma drug than was previously observed for the agent in sarcoma and kidney cancer cells. The [pazopanib + AR42] combination at ∼40–50% of their individual C max values caused significantly more *in vitro* melanoma cell killing, including killing vemurafenib resistant and trametinib/dabrafenib resistant cells, than did the standard of care combination of trametinib/dabrafenib each at their established full C max values. In an animal model system using trametinib/dabrafenib resistant melanoma cells, AR42 as a single agent could significantly retard melanoma tumor growth and prolong animal survival. Exposure of trametinib/dabrafenib resistant tumors to the [pazopanib + AR42] combination for three days significantly reduced tumor growth below baseline levels for two weeks and further extended animal survival beyond that afforded by AR42 as a single agent.

**Figure 14:**
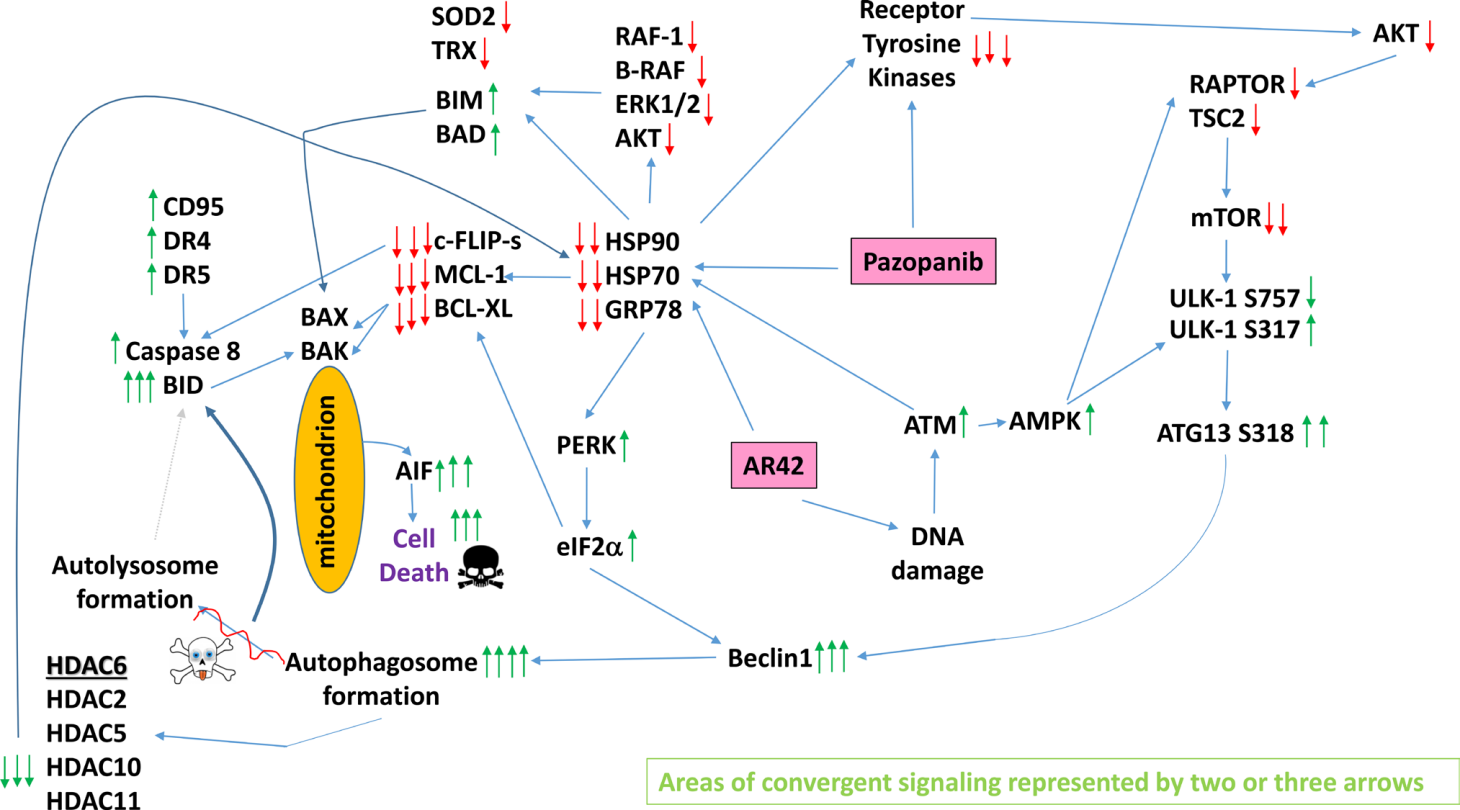
A simplified model of the molecular pathways by which pazopanib and AR42 combine to kill cancer cells As an HDAC inhibitor, AR42 causes DNA damage and causes inhibitory chaperone acetylation. Pazopanib, as a multi-kinase and chaperone inhibitor, also inhibits chaperone activities as well as many class III receptor tyrosine kinases. DNA damage causes activation of ATM. ATM signals to activate the AMPK. AMPK signaling inactivates RAPTOR and TSC2 resulting in the inactivation of mTORC1 and mTORC2. Downstream of mTOR is the kinase ULK-1; the drug combination via AMPK promotes ULK-1 S317 phosphorylation which activates the kinase; the drug combination via mTOR inactivation reduces ULK-1 S757 phosphorylation which also activates the kinase. Activated ULK-1 phosphorylates ATG13 which is the key gate-keeper step in permitting autophagosome formation. AR42-induced ATM signaling also acts to reduce the activities of multiple chaperone proteins. Reduced HSP90 and HSP70 function lowers the expression of all receptor tyrosine kinases and the activities of STAT3, STAT5, ERK1/2 and AKT that results in lower expression of ROS/RNS detoxifying enzymes such as TRX and SOD2. Reduced GRP78 function causes activation of PERK and subsequently eIF2α. Enhanced eIF2α signaling reduces the transcription of proteins with short half-lives such as c-FLIP-s, MCL-1 and BCL-XL, and enhances expression of Beclin1, DR4 and DR5. Thus the convergent actions of reduced HSP90 and HSP70 chaperone activity and eIF2α signaling lead to a profound reduction in the protein levels of c-FLIP-s, MCL-1 and BCL-XL which facilitates death receptor signaling through CD95, DR4 and DR5 to activate the extrinsic apoptosis pathway. Enhanced Beclin1 expression converges with elevated ATG13 phosphorylation to produce high levels of autophagosome formation that acts to reduce HDAC2/5/6/10/11 expression but also to stall autophagosome fusion with lysosomes and to stall autolysosome maturation, which likely through cytosolic cathepsin proteases converges with the extrinsic apoptosis pathway to cleave BID and cause mitochondrial dysfunction. Tumor cell killing downstream of the mitochondrion was mediated by AIF and not caspases 3/7. The tumoricidal actions of AIF were facilitated by reduced HSP70 functionality as this chaperone can sequester AIF in the cytosol and prevent its translocation to the nucleus.

The molecular mechanisms by which pazopanib and AR42 interacted to cause tumor cell death were complex, with cell death being mediated through multiple overlapping and congruent mechanisms (Figure 14). The drug combination: a) activated death receptor signaling through caspase 8 causing mitochondrial dysfunction; b) inhibited the functionality of multiple chaperones through inhibition of their ATPase activities which activated endoplasmic reticulum stress signaling that facilitated both autophagosome formation and suppressed the expression of protective proteins including c-FLIP-s, MCL-1 and BCL-XL; c) caused a DNA damage response which regulated AMPK-dependent mTOR inactivation and induction of toxic autophagosomes as well as toxic NFκB signaling; d) via ATM-AMPK signaling which reduced expression of HDAC6 leading to sustained HSP90 acetylation which will facilitate the ATPase inhibitory effect of pazopanib. All of these mechanisms converged to cause a profound level of mitochondrial dysfunction, likely reducing cellular ATP levels, as killing was mediated by the necroptotic actions of AIF rather than the ATP-dependent mechanism through caspases 9 and 3. Our studies, in addition to PDX melanoma isolates, also examined PDX head & neck carcinoma and ovarian isolates; NSCLC cells; triple negative breast cancer cells; and bladder cancer cells. In all of the tumor cell types tested, AR42 treatment caused significant amounts of tumor cell killing as a single agent and interacted with pazopanib to further enhance killing.

There are a number of recognized mechanisms by which mutant B-RAF melanoma cells evolve under therapeutic pressure to become trametinib/dabrafenib resistant. One evolutionary mechanism, that will bypass the trametinib/dabrafenib drug combination, is the re-activation of ERK1/2 in a manner independent of RAF-MEK signaling; another evolutionary pathway can be through activation of ERBB1; and another by activating the PI3K pathway, e.g. loss of PTEN. We found that the [pazopanib + AR42] combination killed trametinib/dabrafenib -resistant MEL28 cells more effectively that it did parental wild type MEL28 cells. The [pazopanib + AR42] combination also caused high levels of killing in the MEL24 line that expresses mutant B-RAF and lacks PTEN expression as well as in the TPF-12-293 PDX isolate that is vemurafenib resistant. Neither pazopanib nor AR42 are direct inhibitors of the ERK1/2/PI3K/STAT3 pathways and as individual agents neither drug caused a significant decline in ERK1/2 or AKT or STAT3 phosphorylation, whereas the combination of the drugs caused almost complete inactivation of ERK1/2, AKT, STAT3, mTORC1 and mTORC2. Using molecular tools, re-activation of either ERK1/2, AKT, STAT3 or mTOR strongly suppressed drug combination killing. These findings argue that efficient melanoma cell killing in our system requires dysregulation of multiple survival signaling modules, such as ERK-BAD/BIM and AMPK-mTOR-autophagy and ERK-STAT3-MCL-1, shifting their activities/functions from maintaining cell viability to permitting tumor cell death. The most likely explanation why [pazopanib + AR42] has so many pleiotropic toxic effects and inactivates so many cyto-protective pathways is that it inactivates HSP90 family, HSP70 family and particularly the ER localized HSP70 family chaperone GRP78; chaperones essential to maintain ERK1/2, AKT, STAT3 and mTOR activity and essential to keep PERK-eIF2α signaling dormant. Collectively, this data suggests that by disregarding the in vogue approach of using highly specific kinase inhibitors for personalized medicine approaches against a specific clonal tumor cell type, e.g. the dabrafenib/trametinib combination against mutant B-RAF, we have been able to out-fox both the evolutionary survival responses of tumor cells as well as being able to kill tumor cells carrying a wide variety of upstream mutations that would predict for drug resistance and a shorter overall patient survival.

Our data demonstrating that AR42, in addition to being an HDAC inhibitor, can also rapidly reduce the expression of HDACs 2/5/6/10/11 was unexpected. The ability of AR42 to down-regulate HDAC6 protein levels was similar to that exhibited by valproate. It is already known that HDAC6 in complex with other proteins can regulate the initial stages of autophagosome formation and can facilitate the appropriate localization of ubiquinated proteins for their degradation [27, 28, 30, 41–43]. And, furthermore, that the AMPK can phosphorylate and destabilize HDAC6, with its degradation occurring through ubiquitination. Some of these analyses, however, over-expressed HDAC proteins whereas our studies have examined endogenous protein levels [44]. In our present studies, although the AMPK was partially activated by AR42, HDAC6 degradation was not blocked by the E3 ligase inhibitor bortezomib. Instead, inhibition of autophagosome formation by knocking down either ATG5 or Beclin1 prevented the AR42-mediated reduction in HDAC6 levels. In contrast to these findings, we also found that the expression levels of LAMP2 and p62/SQSMT1 were elevated in [pazopanib + AR42] treated cells which argues lysosomal digestive functions are reduced by the drug combination. Yet HDAC6 degradation was lysosomal. HDAC10 is essential for autophagosome – lysosome fusion as well as autolysosome maturation. Collectively our data argues that in our drug-combination system HDAC6 and HDAC10 play an essential role in both the fusion of autophagosomes with lysosomes as well as the maturation of autolysosomes. In cells lacking HDAC6, [pazopanib + AR42] could not further increase the levels of p62 but did so for LAMP2 arguing that [pazopanib + AR42] through HDAC6-dependent and HDAC6-independent mechanisms regulates autolysosome maturation i.e. a requirement for HDAC10. HDAC6 degradation was co-localized with LAMP2 validating the concept of HDAC6 degradation in the lysosome. As we had also found in our system that AMPK signaling was essential for autophagosome formation upstream of mTOR, we are thus at present unsure whether the actions of the AMPK regulate HDAC6 expression levels through direct phosphorylation or indirectly through modulation of mTOR and autophagy. Studies beyond the scope of the present manuscript will be required to understand the complexity of HDAC6 expression/activity regulation/lysosomal protease activity by AR42.

Although the combination of trametinib/dabrafenib has considerably prolonged disease free progression and overall survival for patients with mutant B-RAF melanoma, eventually tumor cells under the restrictive pressure of the drug combination evolve mechanisms of resistance. To determine in a side-by-side fashion the relative effectiveness of trametinib/dabrafenib, pazopanib, AR42 and [pazopanib + AR42] we performed *in vivo* studies using MEL28 cells made resistant by *in vitro* culture with trametinib/dabrafenib. Animals were treated for three days with the drugs using a dosing schedule and drug levels approximately equivalent to the standard of care in melanoma and to our on-going phase I trial in sarcoma/RCC combining [pazopanib + AR42]. Treatment of animals for three days with trametinib/dabrafenib initially suppressed tumor growth but over time did not prolong animal survival. Survival for animals treated with trametinib/dabrafenib was enhanced by approximately four days, arguing that the anti-tumor effects of RAF/MEK inhibition were related to growth arrest effects and not to actual tumor cell killing. As single agents both pazopanib and AR42 significantly enhanced animal survival, each by approximately 8 days, arguing that both growth arrest and tumor cell killing effects were occurring. The combination of [pazopanib + AR42] caused a further significant enhancement in animal survival over that afforded by either pazopanib or AR42 alone, and that was associated with an initial prolonged reduction in tumor mass below pre-treatment levels. Collectively, the *in vivo* data using trametinib/dabrafenib resistant MEL28 cells argues that the combination of pazopanib with AR42, or with sodium valproate, should be explored as a new phase I/II trial in mutant B-RAF melanoma patients.

In prior studies we have performed antibody array multiplex analyses to define biomarkers in tumors and animal plasma previously exposed to various drug combination [4, 11, 39]. In the present studies we discovered that transient HDAC inhibitor treatment of melanoma tumors resulted in several profound evolutionary changes in tumor cell biology. Notably, AR42 doubled the already high expression of human PDGF in mouse plasma, a compensatory effect that may explain why the phase I trial in melanoma using panobinostat as a single agent failed; whatever anti-tumor effects panobinostat may have exhibited would have been swamped by the compensatory induction of PDGF expression. In addition, the post-therapy plasma levels of bFGF, HGF, G-CSF and prolactin were also elevated. Pazopanib is a recognized inhibitor of VEGFR1-3, FGF family receptors, PDGFRα/β, c-KIT and FMS. Thus under a treatment protocol with AR42 where pazopanib is dosed daily, the evolution of elevated levels of PDGF and bFGF will be counteracted by the kinase-inhibitory actions of pazopanib. Pazopanib does not block signaling by c-MET, SRC family kinases or Janus kinases, enzymes predicted to be activated by HGF, G-CSF and prolactin. We discovered that inhibition of MET-STAT3 signaling could enhance [pazopanib + AR42] lethality. Future studies focused on the MET-STAT3 axis will be required to fully define the complex signaling network by which [pazopanib + AR42] treated cells evolve and survive. The impact of altered cytokine expression in the evolved surviving tumors will required studies beyond the scope of the present manuscript.

## MATERIALS AND METHODS

### Materials

Pazopanib and AR42 and other kinase inhibitors were purchased from Selleckchem (Houston, TX). Sodium valproate was from Sigma (St. Louis, MO). Trypsin-EDTA, DMEM, RPMI, penicillin-streptomycin were purchased from GIBCOBRL (GIBCOBRL Life Technologies, Grand Island, NY). J28, T24, MEL24, MEL28, OVCAR, PAI, SKOV3, HCC38, SUM149, A549, H460 and H1975 cells were purchased from the ATCC and were not further validated beyond that claimed by ATCC. Cells were re-purchased every ∼6 months. Characterized PDX melanoma isolates expressing mutated active B-RAF were from the University of Pittsburgh's melanoma cell bank. H&N PDX tumor isolates were kindly provided by Dr. Lee. ADOR cells were a gift to the Dent lab from a female NSCLC patient. Spiky, CTG-1703, CTG-1677 PDX ovarian cancer cells were provided by Dr. Karen Paz (Champions Oncology, NJ). Commercially available validated short hairpin RNA molecules to knock down RNA/protein levels were from Qiagen (Valencia, CA) (Supplementary Figures 24 and 25). Reagents and performance of experimental procedures were described in refs: [1–8].

### Methods

### Culture and *in vitro* exposure of cells to drugs

All cell lines were cultured at 37°C (5% (v/v CO_2_) *in vitro* using RPMI supplemented with dialyzed 5% (v/v) fetal calf serum and 10% (v/v) Non-essential amino acids. *In vitro* drug treatments were generally from a 100 mM stock solution of each drug and the maximal concentration of Vehicle carrier (VEH; DMSO) in media was 0.02% (v/v). Cells were not cultured in reduced serum media during any study in this manuscript. Unless otherwise stated, cells were treated with vehicle control, pazopanib (1 μM), AR42 600 nM, or the drugs in combination for 6 h–24 h. Where indicated cells were also treated with trametinib (2.0 μM) and dabrafenib (2.0 μM) combined; MEL28-R cells were generated by being grown in the presence of trametinib (2.0 μM) and dabrafenib (2.0 μM) for 14 days. Surviving cells were collected and 14 days later re-exposed to the drugs for 14 days. For cell death analyses, at the indicated time point floating cells were cyto-spun onto the 96 well plate and cell viability determined using a live/dead viability stain. For immuno-fluorescence studies cells are fixed in place and permeabilized using ice cold PBS containing 0.4% paraformaldehyde and 0.5% Triton X-100.

### Transfection of cells with siRNA or with plasmids

For Plasmids: Cells were plated and 24 h after plating, transfected. Plasmids expressing a specific mRNA (or siRNA) or appropriate vector control plasmid DNA was diluted in 50 μl serum-free and antibiotic-free medium (1 portion for each sample). Concurrently, 2 μl Lipofectamine 2000 (Invitrogen), was diluted into 50 μl of serum-free and antibiotic-free medium (1 portion for each sample). Diluted DNA was added to the diluted Lipofectamine 2000 for each sample and incubated at room temperature for 30 min. This mixture was added to each well/dish of cells containing 200 μl serum-free and antibiotic-free medium for a total volume of 300 μl, and the cells were incubated for 4 h at 37°C. An equal volume of 2× medium was then added to each well. Cells were incubated for 24 h, then treated with drugs.

### Transfection for siRNA

Cells from a fresh culture growing in log phase as described above, and 24 h after plating transfected. Prior to transfection, the medium was aspirated and serum-free medium was added to each plate. For transfection, 10 nM of the annealed siRNA, the positive sense control doubled stranded siRNA targeting GAPDH or the negative control (a “scrambled” sequence with no significant homology to any known gene sequences from mouse, rat or human cell lines) were used. Ten nM siRNA (scrambled or experimental) was diluted in serum-free media. Four μl Hiperfect (Qiagen) was added to this mixture and the solution was mixed by pipetting up and down several times. This solution was incubated at room temp for 10 min, then added drop-wise to each dish. The medium in each dish was swirled gently to mix, then incubated at 37°C for 2 h. Serum-containing medium was added to each plate, and cells were incubated at 37°C for 24 h before then treated with drugs (0–24 h). Additional immuno-fluorescence/live-dead analyses were performed at the indicated time points.

### Animal studies

Studies were performed according to USDA regulations under VCU IACUC protocol AD20008. Athymic male nude mice (∼20 g) were injected with 1 × 10^6^ Trametinib/Dabrafenib resistant MEL28 cells (MEL28-R cells) into their rear flank (10 animals per treatment group; 5 groups; a total of 50 mice). The SK-MEL-28 line was established from an axillary lymph node of a 51-year-old male of unknown ethnicity. Tumors were permitted to form for 7 days with tumors at that time exhibiting a mean volume of 9.0 mm^3^. Mice were treated for 3 days by oral gavage with vehicle control, trametinib (30 mg/kg), dabrafenib (2.0 mg/kg), pazopanib (20 mg/kg), AR42 (15 mg/kg, Days 1 and 3) or the drugs in combination, as indicated. Before, during and after drug treatment tumors are calipered as indicated in the Figure and tumor volume was assessed up to 20–40 days later. The -Fold increase in tumor volume is plotted in Figure 13A. When the volume of the tumor reached > 250 mm^3^, (equivalent of a 0.75 kg tumor in a 60 kg person), animals were humanely sacrificed and the tumor and blood removed for further studies. Animal survival was plotted on a Kaplan–Meier graph and longitudinal statistical assays performed to determine whether any changes were significant (Figure 13B).

### Detection of cell viability, protein expression and protein phosphorylation by immuno-fluorescence using a hermes WiScan machine

http://www.idea-bio.com/, Cells (4 × 10^3^) are plated into each well of a 96 well plate, and cells permitted to attach and grow for the next 18 h. Based on the experiment, after 18 h, cells are then either genetically manipulated, or are treated with drugs. For genetic manipulation, cells are transfected with plasmids or siRNA molecules and incubated for an additional 24 h. Cells are treated with vehicle control or with drugs at the indicated final concentrations, alone or in combination. Cells are then isolated for processing at various times following drug exposure. The 96 well plate is centrifuged/cyto-spun to associate dead cells (for live-dead assays) with the base of each well. For live dead assays, after centrifugation, the media is removed and cells treated with live-dead reagent (Thermo Fisher Scientific, Waltham MA) and after 10 min this is removed and the cells in each well are visualized in the Hermes instrument at 10× magnification. Green cells = viable; yellow/red cells = dying/dead. The numbers of viable and dead cells were counted manually from three images taken from each well combined with data from another two wells of separately treated cells (i.e. the data is the mean cell dead from 9 data points from three separate exposures). For immuno-fluorescence studies, after centrifugation, the media is removed and cells are fixed in place and permeabilized using ice cold PBS containing 0.4% paraformaldehyde and 0.5% Triton X-100. After 30 min the cells are washed three times with ice cold PBS and cells are pre-blocked with rat serum for 3 h. Cells are then incubated with a primary antibody to detect the expression/phosphorylation of a protein (usually at 1:100 dilution from a commercial vendor) overnight at 37^°^C. Cells are washed three times with PBS followed by application of the secondary antibody containing an associated fluorescent red or green chemical tag. After 3 h of incubation the antibody is removed and the cells washed again. The cells are visualized at either 10× or 60× in the Hermes machine for imaging assessments. All immunofluorescent images for each individual protein/phospho-protein are taken using the identical machine settings so that the levels of signal in each image can be directly compared to the level of signal in the cells treated with drugs. Changes in immunofluorescence intensity are determined using the Hermes's integral imaging software from forty cell images taken from each well combined with data from another two wells of separately treated cells (i.e. the data is the mean cell dead from 120 cell imaging data points from three separate exposures). Similarly, for presentation, the enhancement of image brightness/contrast using PhotoShop CS6 is simultaneously performed for each individual set of protein/phospho-protein to permit direct comparison of the image intensity between treatments. Antibodies used include: HSP90 (E289) (Cell Signaling); HSP90 (#2928) (Abcam); HSP90 (ab195575) Abcam; HSP90 3G3 (13495) (Abcam); GRP78 (50b12) (31772) (Cell Signaling); GRP78 (ab191023) Abcam; GRP78 (ab103336) Abcam; GRP78 (N-20) (sc-1050) Santa Cruz; HSP27 (G31) (2402P) Cell Signaling); HSP27 [EP1724Y] (ab62339) Abcam; HSP27 (H-77) (sc-9012) Santa Cruz; HSP27 (LS-C31836) Lifespan science Corp. Other antibodies were as used in prior studies by the laboratory. All immunofluorescent images were initially visualized at 75 dpi using an Odyssey infrared imager (Li-Cor, Lincoln, NE), then processed at 9999 dpi using Adobe Photoshop CS6. For presentation, immunoblots were digitally assessed using the provided Odyssey imager software. Images have their color removed and labeled figures generated in Microsoft PowerPoint.

### Assessment of autophagy

Cells were transfected with a plasmid to express a green fluorescent protein (GFP) and red fluorescent protein (RFP) tagged form of LC3 (ATG8). For analysis of cells transfected with the GFP-RFP-LC3 construct, the GFP/RFP-positive vesicularized cells were examined under the ×40 objective of a Zeiss Axiovert fluorescent microscope. At each time point the mean number of intense GFP/RFP-LC3 punctae per cell was determined from at least 40 cells per condition.

### Multiplex assays

Were performed as previously described [4]: bio-plex-pro-human-cancer-biomarker-panel-1-16-plex; bio- plex-pro-human-cancer-biomarker-panel-2-18-plex; bio- plex-pro-human-inflammation-panel-1-37-plex; bio-plex- pro-cell-signaling-mapk-panel-9-plex; bio-plex-pro-cell -signaling-akt-panel-8-plex; bio-plex-pro-phospho-egfr- tyr1068-set; bio-plex-pro-phospho-egfr-tyr1173-set; bio- plex-pro-phospho-her-2-tyr1248-set; bio-plex-pro-phospho -ikappab-alpha-ser32-ser36-set; bio-plex-pro-phospho-nf- kappab-p65-ser536-set; bio-plex-pro-phospho-p53-ser15 -set; bio-plex-pro-phospho-pdgfr-alpha-tyr754-set; bio- plex-pro-phospho-pdgfr-beta-tyr751-set; bio-plex-pro-phospho-src-tyr416-set; bio-plex-pro-phospho-stat1-tyr701-set; bio-plex-pro-phospho-stat3-tyr705-set; bio-plex- pro-total-akt-set; bio-plex-pro-total-erk1-2-set; bio-plex-pro-total-pten-set; bio-plex-pro-total-ikappab-alpha-set.

### Chaperone activity assays

Cells were transfected with plasmids to express either FLAG-HSP90 or HA-HSP70 or FLAG-GRP78. Twenty-four h after transfection cells were treated with drugs; vehicle control, AR42 (600 nM). One hour later the cells were lysed and the HSP90 and HSP70 and GRP78 immunoprecipitated using their tags. Chaperone ATPase activity using the ATPlite 1step kit (PerkinElmer) was determined using the chaperones still linked to the Sepharose 4B beads. The Sepharose beads were equilibrated in the reaction buffer provided by the manufacturer for 30 min with gentle mixing, and the beads recovered by centrifugation. The beads were then resuspended 1:1 with reaction buffer. To each well of a 96 well plate was added 50 μl of bead slurry and 50 μl of substrate buffer solution containing vehicle control or drug to achieve the desired final drug concentrations. The reactions were started using a multi-channel pipette delivering 50 μl of reconstituted reagent to each well. The plate was placed in foil in an orbital shaker at 37°C for 15 min. The plate was removed, centrifuged to remove floating Sepharose beads, and 100 μl of the supernatant from each well placed into a new well in another 96 well plate. The light emitted from each well/treatment condition was quantified using a Vector 3 plate reader (*n* = 3 of three studies +/− SEM).

### Data analysis

Comparison of the effects of various treatments (performed in triplicate three times) was using one-way analysis of variance and a two tailed Student's *t-test*. Statistical examination of *in vivo* animal survival data utilized both a two tailed Student's *t-test* and log rank statistical analyses between the different treatment groups. Differences with a *p-value* of < 0.05 were considered statistically significant. Experiments shown are the means of multiple individual points from multiple experiments (± SEM).

## SUPPLEMENTARY MATERIALS FIGURES


