# Supplementary material for: The HDAC inhibitor AR42 interacts with pazopanib to kill trametinib/dabrafenib-resistant melanoma cells in vitro and in vivo
Source: Oncotarget. 2017 Jan 27;8(10):16367–86. doi: 10.18632/oncotarget.14829 (PMC5369969; doi:10.18632/oncotarget.14829)
Supplement: Supplementary file 1 [file oncotarget-08-16367-s001.pdf]

The HDAC inhibitor AR42 interacts with pazopanib to kill trametinib/dabrafenib-resistant melanoma cells *in vitro* and *in vivo*

Supplementary Materials

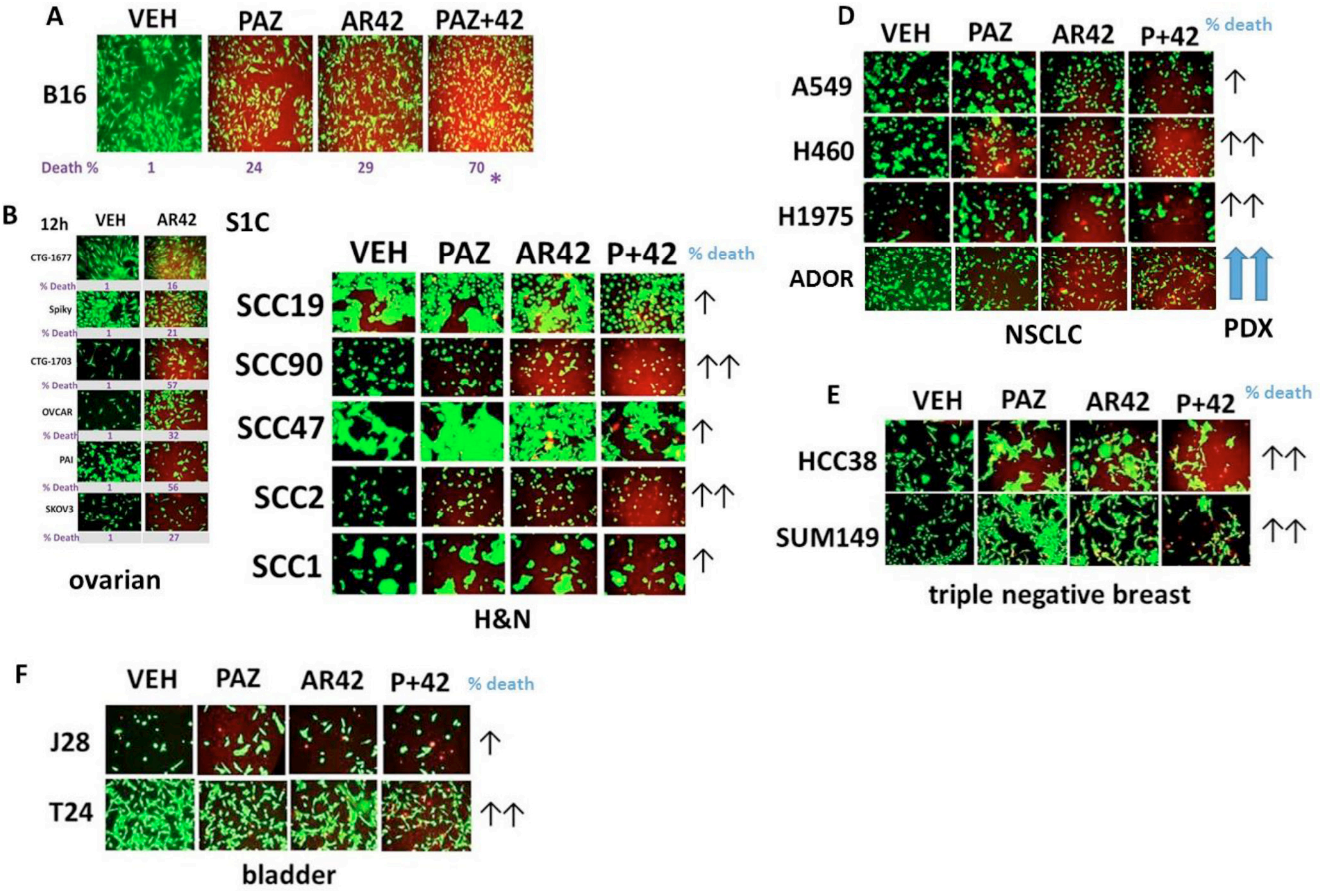

Supplementary Figure 1: Pazopanib and AR42 interact to kill multiple diverse types of tumor cell. (**A**) B16 mouse melanoma cells and additional human PDX isolates were treated with vehicle control, pazopanib (1 µM), AR42 (600 nM) or the drugs in combination for 24 h. Floating cells were cyto-spun onto the 96 well plate and cell viability determined using a live/dead viability stain (n = 3 +/– SEM). *$p < 0.05$ greater than either pazopanib alone or AR42 alone values. (**B–F**) Tumor cells were treated with vehicle control, pazopanib (1 µM), AR42 (600 nM) or the drugs in combination for 24 h. Floating cells were cyto-spun onto the 96 well plate and cell viability determined using a live/dead viability stain.

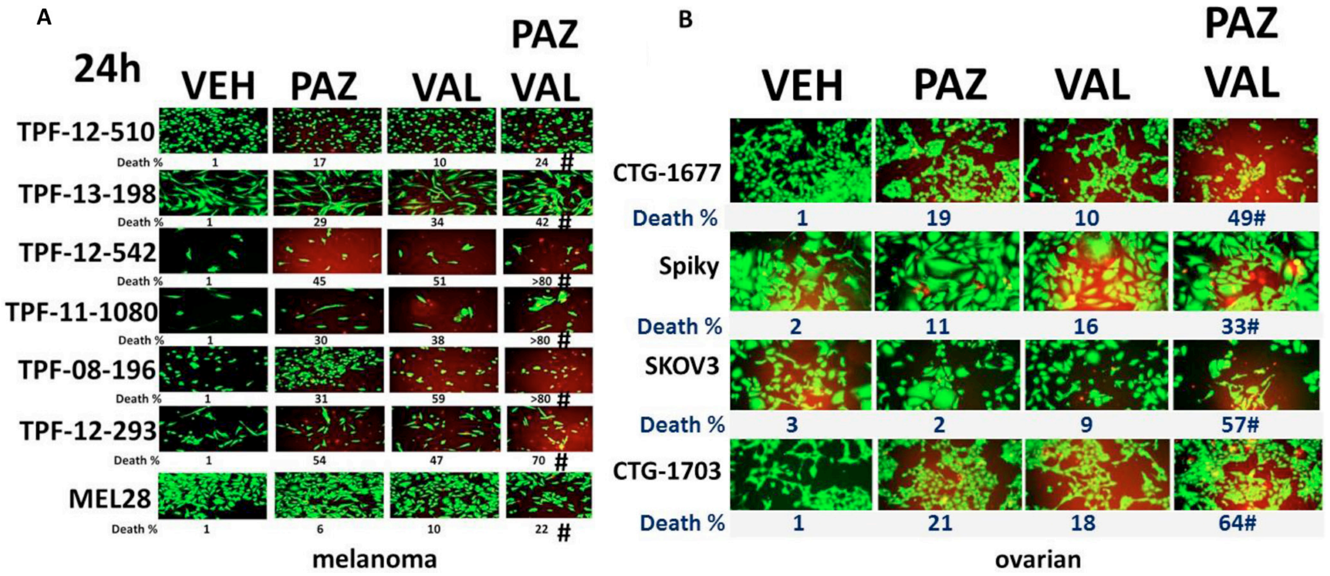

Supplementary Figure 2: Pazopanib and sodium valproate interact to kill melanoma and ovarian cancer cells. (**A**) Melanoma cells and (**B**) ovarian cancer cells were treated with vehicle control, pazopanib (1 μM), sodium valproate (250 μM) or the drugs in combination for 24 h. Floating cells were cyto-spun onto the 96 well plate and cell viability determined using a live/dead viability stain (n = 3 +/- SEM). $^{\#}$ p < 0.05 greater than either pazopanib alone or AR42 alone values.

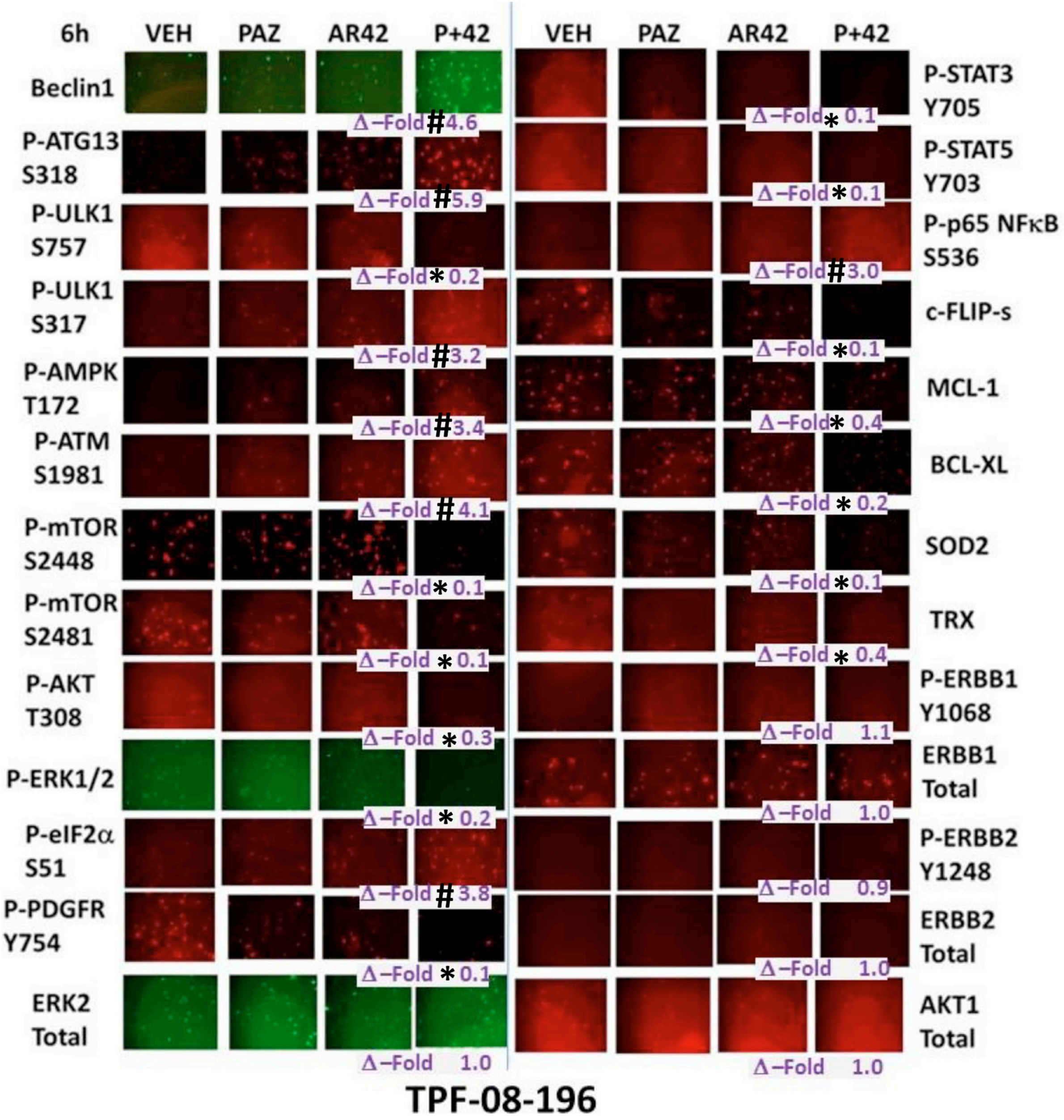

Supplementary Figure 3: Pazopanib and AR42 interact to modulate the expression and phosphorylation of multiple intracellular proteins. TPF-08-196 cells were treated with vehicle control, pazopanib (1.0 µM), AR42 (600 nM) or the drugs in combination for 6 h. Cells were then fixed in place with permeabilization. Immuno-fluorescence was performed at 10X magnification in a Hermes wide field microscope to determine the gross changes in the phosphorylation and total expression of the indicated proteins (representative images are shown, $n = 3$ +/- SEM). $^{\#}p < 0.05$ greater than either pazopanib alone or AR42 alone values. $^{*}p < 0.05$ less than either pazopanib alone or AR42 alone values.

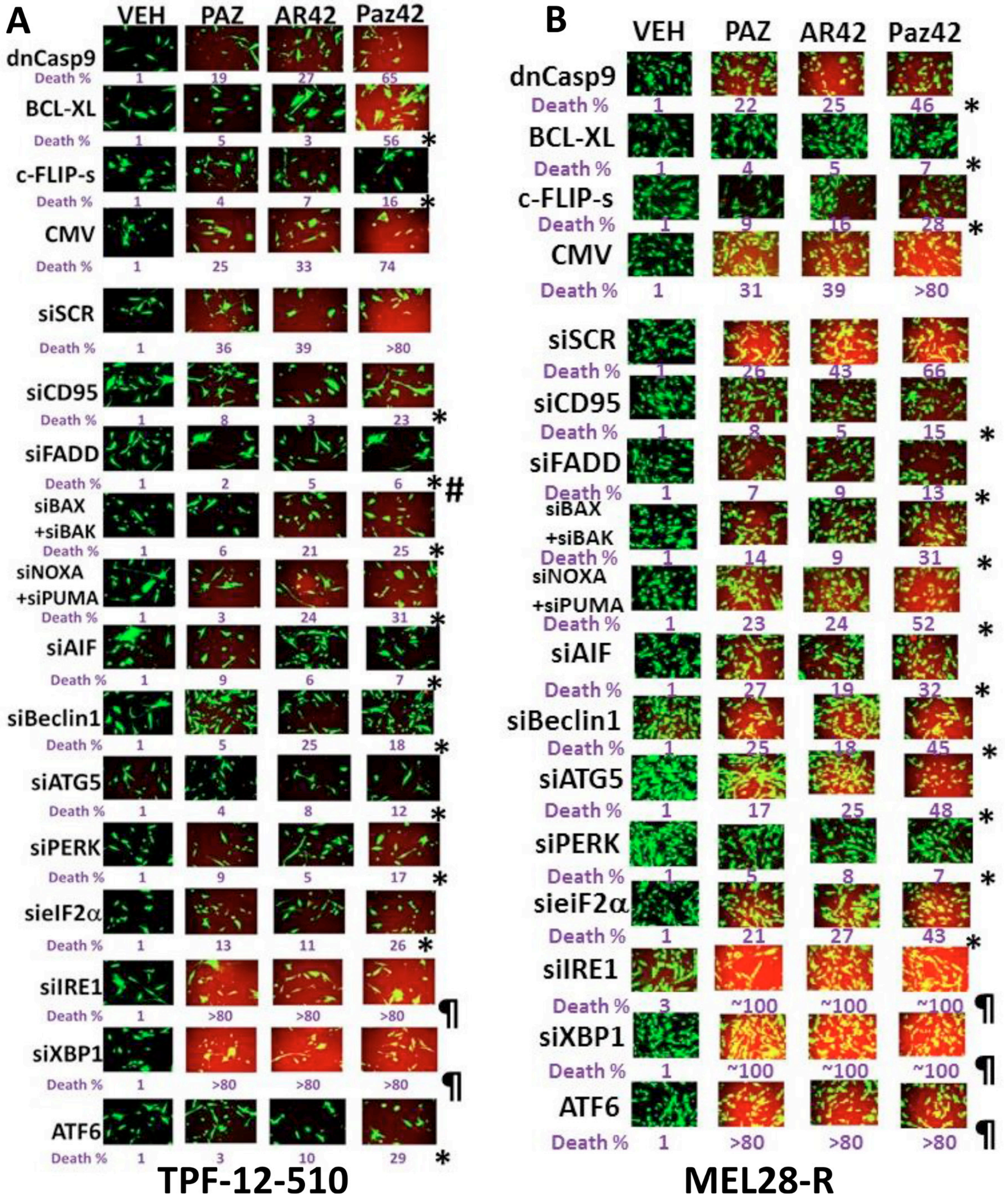

Supplementary Figure 4: Pazopanib and AR42 interact to kill melanoma cells through death receptor signaling; autophagy; and ER stress signaling. TPF-12-510 and MEL28-R cells were either: transfected with an empty vector plasmid (CMV) or with plasmids to express dominant negative caspase 9, BCL-XL or c-FLIP-s; or with a scrambled siRNA (siSCR) or with siRNA molecules to knock down the indicated proteins. Twenty-four h after transfection cells were treated with vehicle control, pazopanib (1 μM), AR42 (600 nM) or the drugs in combination for 24 h. Floating cells were cyto-spun onto the 96 well plate and cell viability determined using a live/dead viability stain (n = 3 +/- SEM). ¶$p < 0.05$ greater than corresponding value in siSCR transfected cells; *$p < 0.05$ less than corresponding value in siSCR cells $^{\#}$ $p < 0.05$ less than corresponding value in siCD95 cells.

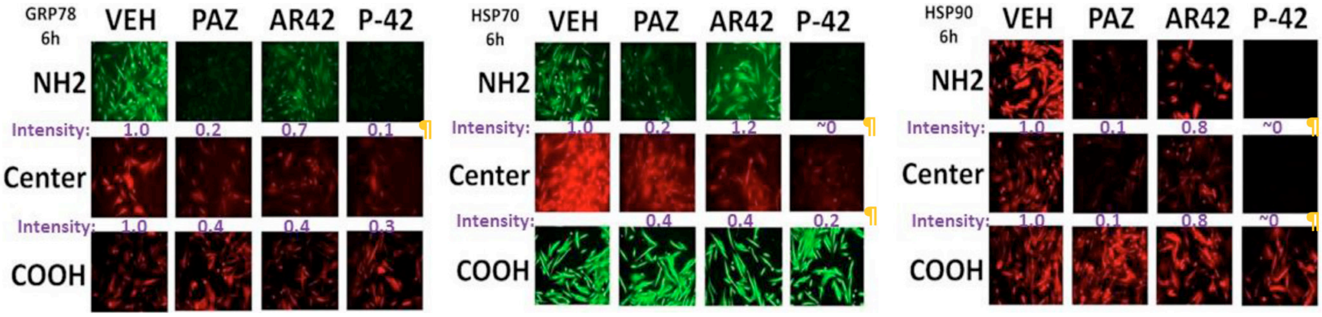

Supplementary Figure 5: AR42 facilitates pazopanib binding to the NH2-termini of chaperone proteins. TPF-12-293 cells were treated with vehicle control, pazopanib (1 μM), AR42 (600 nM) or the drugs in combination for 6 h. Cells were fixed in place and permeabilized, and immuno-fluorescence performed to detect the expression of the indicated proteins; multiple antibodies were used to detect the expression of each chaperone with epitopes in the NH2-terminus, the central portion of the protein and the COOH-terminus (n = 3 +/- SEM) ¶$p < 0.05$ less than value in pazopanib alone.

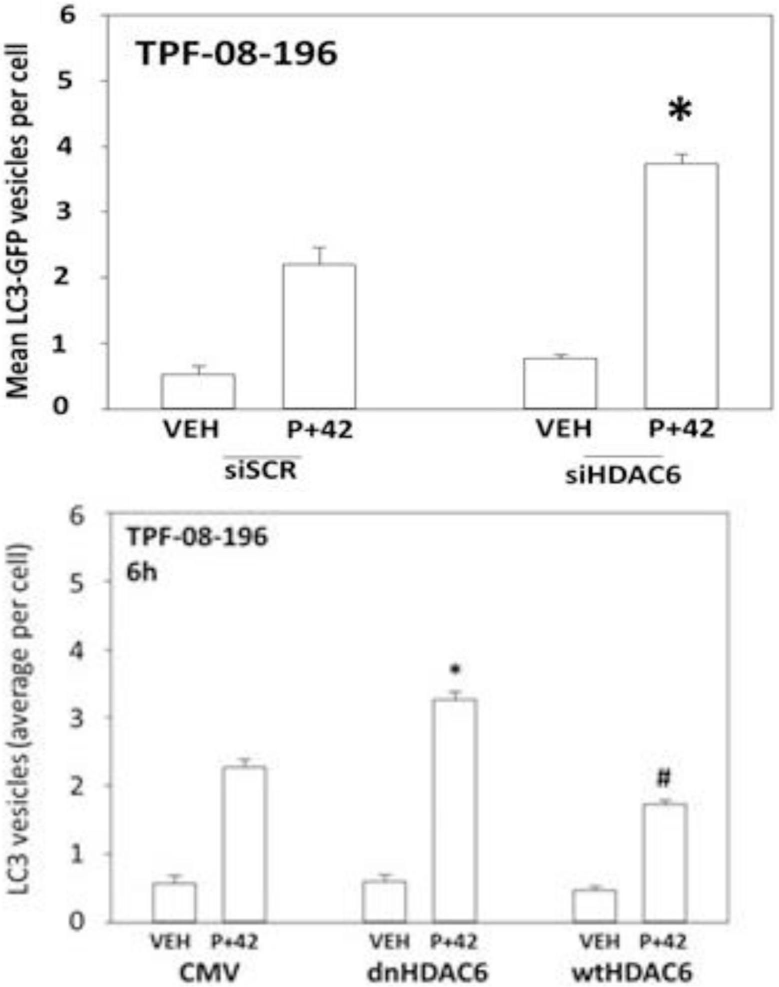

Supplementary Figure 6: HDAC6 regulates the induction of autophagosomes by [pazopanib + AR42]. TPF-08-196 melanoma cells were transfected with a plasmid to express LC3-GFP and in parallel co-transfected with either an empty vector plasmid; a plasmid to express wild type HDAC6; or a plasmid to express dominant negative HDAC6; or transfected with a scrambled siRNA or an siRNA to knock down HDAC6 expression. Twenty-four h after transfection cells were treated with vehicle control or with [pazopanib (1 μM) + AR42 (600 nM)] for 6h. The numbers of punctate LC3-GFP staining vesicles were counted in at least 40 cells per condition and the mean number of vesicles per cell plotted (n = 3 +/- SEM) *$p < 0.05$ greater than corresponding value in CMV/SCR cells; $^{\#}$ $p < 0.05$ less than corresponding value in CMV/SCR cells.

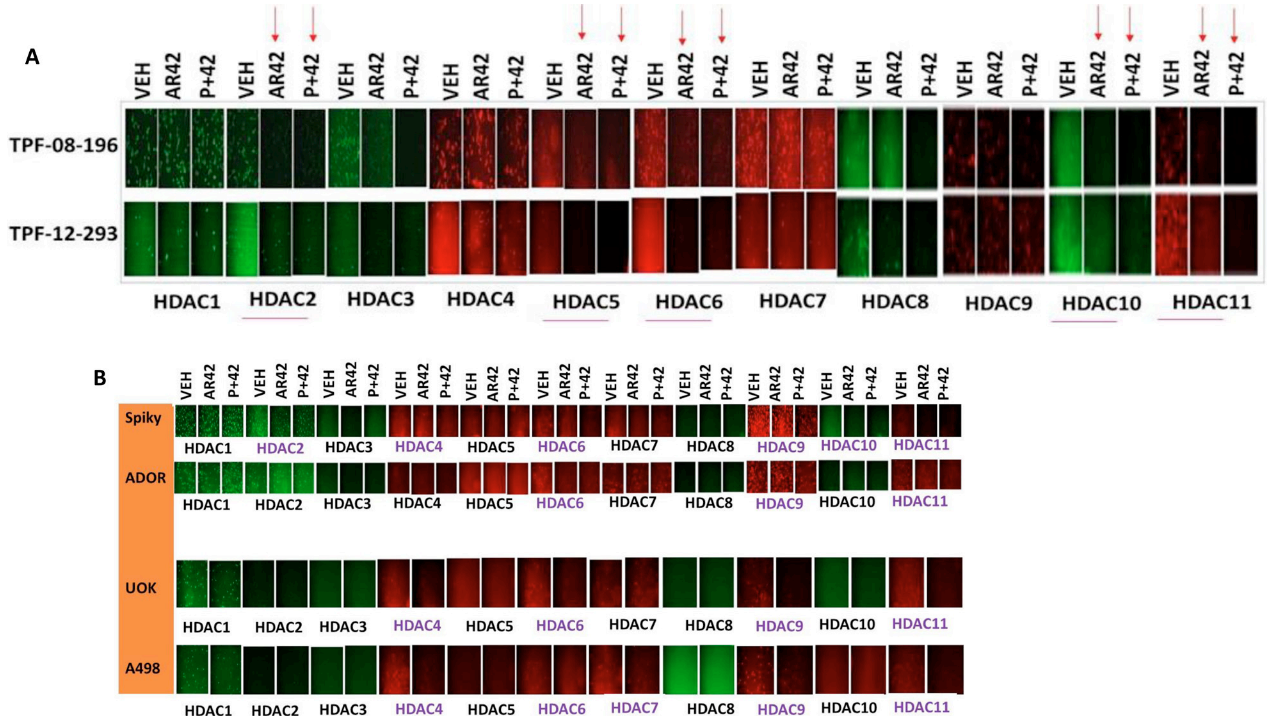

Supplementary Figure 7: AR42 and [pazopanib + AR42] rapidly reduce the expression of HDACs in melanoma, lung, ovarian and renal cancer cells. (**A**) ADOR (PDX NSCLC), Spiky (PDX ovarian) and UOK121LN (PDX renal) cancer cells and A498 established renal carcinoma cells were treated for 6h with vehicle control; AR42 (600 nM); or [pazopanib (1 μM) + AR42]. Cells were then fixed and immunostaining performed to determine the protein expression levels of HDAC1-11. Red arrows indicate a statistically significant reduction in HDAC staining intensity compared to vehicle control in both PDX isolates ($p < 0.05$). (**B**) Melanoma cells were treated for 6h with vehicle control; AR42 (600 nM); or [pazopanib (1 μM) + AR42]. Cells were then fixed and immunostaining performed to determine the protein expression levels of HDAC1-11. Purple text indicates a statistically significant reduction in HDAC staining intensity compared to vehicle control in the PDX isolates ($p < 0.05$).

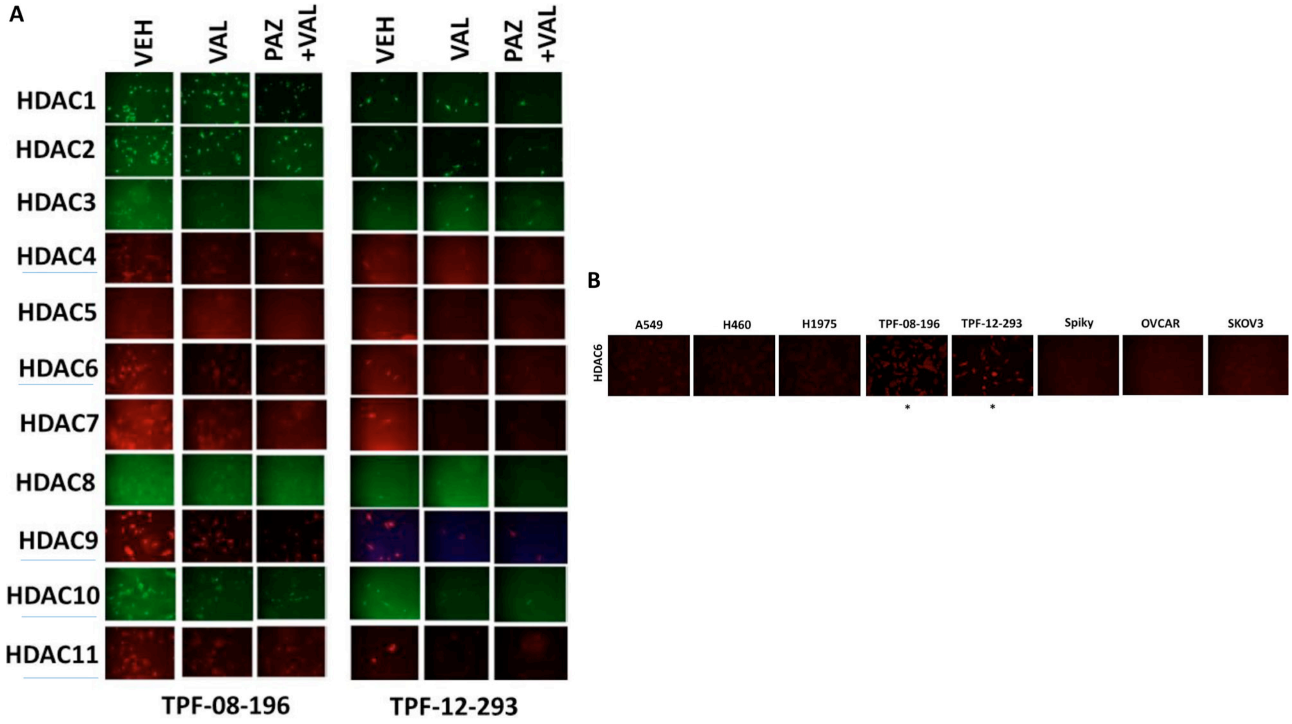

Supplementary Figure 8: Valproate and [pazopanib + valproate] rapidly reduce the expression of HDACs in melanoma cells. (**A**) TPF-08-196 and TPF-12-293 melanoma cells were treated for 6h with vehicle control; sodium valproate (250 μM); or [pazopanib (1 μM) + valproate]. Cells were then fixed and immunostaining performed to determine the protein expression levels of HDAC1-11. Purple text indicates a statistically significant reduction in HDAC staining intensity compared to vehicle control in the PDX isolates ($p < 0.05$). (**B**) Tumor cells were plated and 24h after plating, fixed in place. Staining was performed to determine the basal expression of HDAC6 ($n = 3$ +/-SEM) *$p < 0.05$ greater intensity of staining than in other tumor cell types (melanoma).

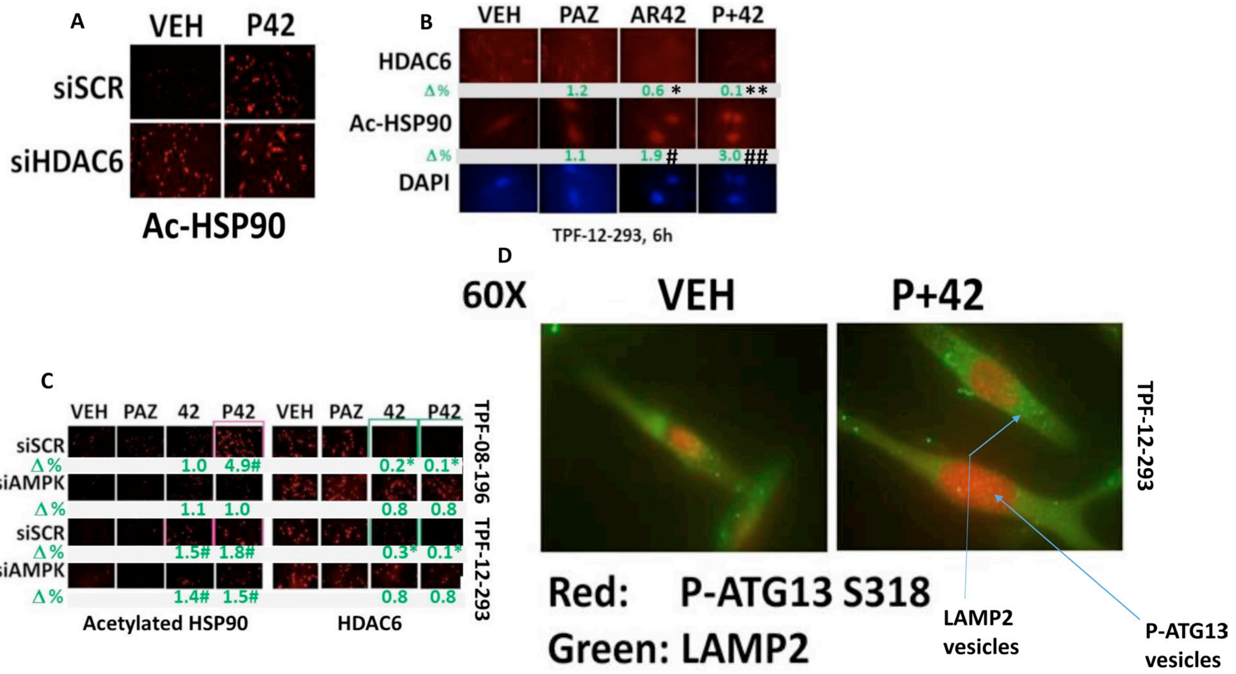

Supplementary Figure 9: HDAC6 regulates the acetylation and expression level of HSP90. (**A**) TPF-12-293 cells were transfected with a scrambled siRNA (siSCR) or an siRNA to knock down the expression of HDAC6. Twenty-four h after transfection, cells were fixed in place and immuno-staining performed to determine the acetylation of HSP90. (**B**) TPF-12-293 cells were treated with vehicle control, pazopanib (1.0 µM), AR42 (600 nM) or the drugs in combination for 6h. Fixed cells were immuno-stained to determine the expression of HDAC6 and the acetylation of HSP90. (n = 3 +/- SEM). $*p < 0.05$ less than vehicle control; $**p < 0.05$ less than AR42 alone value; $^{\#}p < 0.05$ greater than vehicle control value; $^{\#\#}p < 0.05$ greater than AR42 alone value. (**C**) Melanoma cells were transfected with a scrambled siRNA or an siRNA to knock down the alpha subunit of the AMPK. Twenty-four h after transfection cells were treated with vehicle control, pazopanib (1.0 µM), AR42 (600 nM) or the drugs in combination for 6h. Fixed cells were immuno-stained to determine the expression of HDAC6 and the acetylation of HSP90. (n = 3 +/- SEM). $*p < 0.05$ less than vehicle control; $^{\#}p < 0.05$ greater than vehicle control. (**D**) TPF-12-293 cells were treated with vehicle control or with [pazopanib (1 µM) + AR42 (600 nM)] for 6h. Cells were fixed in place and immuno-staining performed to detect the expression and co-localization of P-ATG13 S318 and LAMP2.

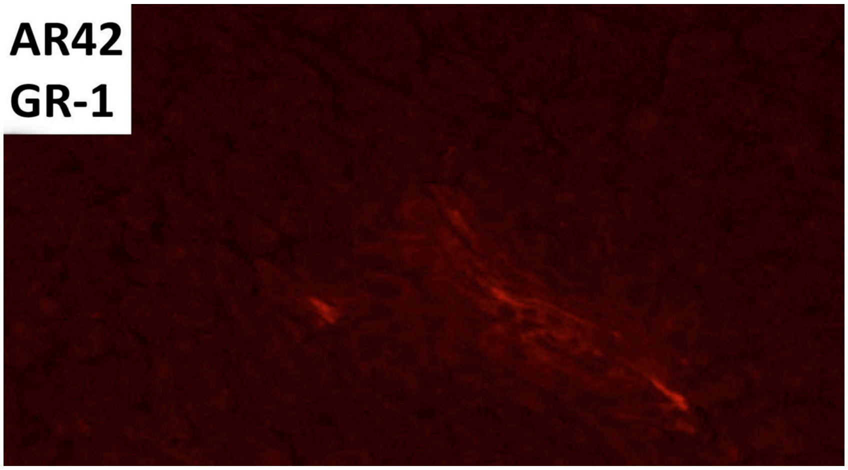

Supplementary Figure 10: Representative enlarged images of AR42 treated and [pazopanib + AR42] treated MEL28-R cells. Four micron slides of AR42 treated and [pazopanib + AR42] tumors were made and immunohistochemistry performed to determine the expression of the indicated proteins.

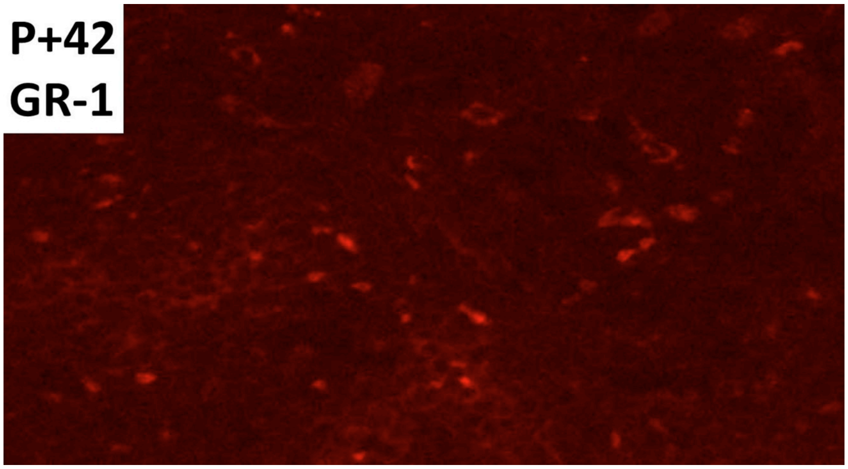

Supplementary Figure 11: Representative enlarged images of AR42 treated and [pazopanib + AR42] treated MEL28-R cells. Four micron slides of AR42 treated and [pazopanib + AR42] tumors were made and immunohistochemistry performed to determine the expression of the indicated proteins.

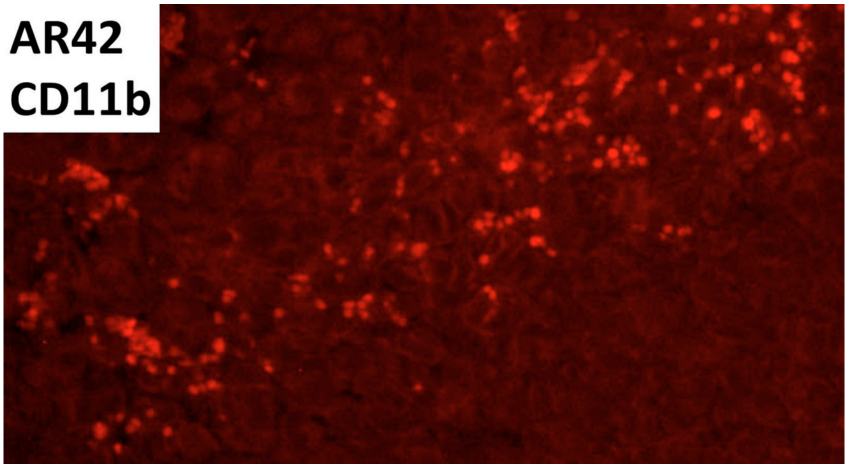

Supplementary Figure 12: Representative enlarged images of AR42 treated and [pazopanib + AR42] treated MEL28-R cells. Four micron slides of AR42 treated and [pazopanib + AR42] tumors were made and immunohistochemistry performed to determine the expression of the indicated proteins.

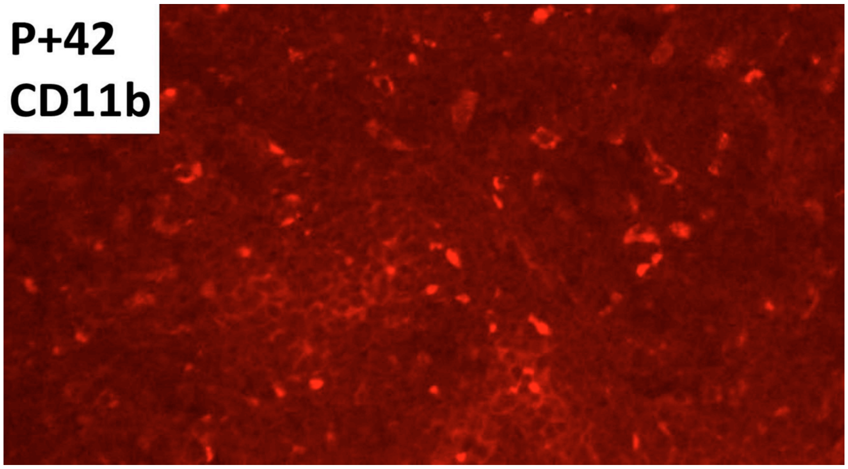

Supplementary Figure 13: Representative enlarged images of AR42 treated and [pazopanib + AR42] treated MEL28-R cells. Four micron slides of AR42 treated and [pazopanib + AR42] tumors were made and immunohistochemistry performed to determine the expression of the indicated proteins.

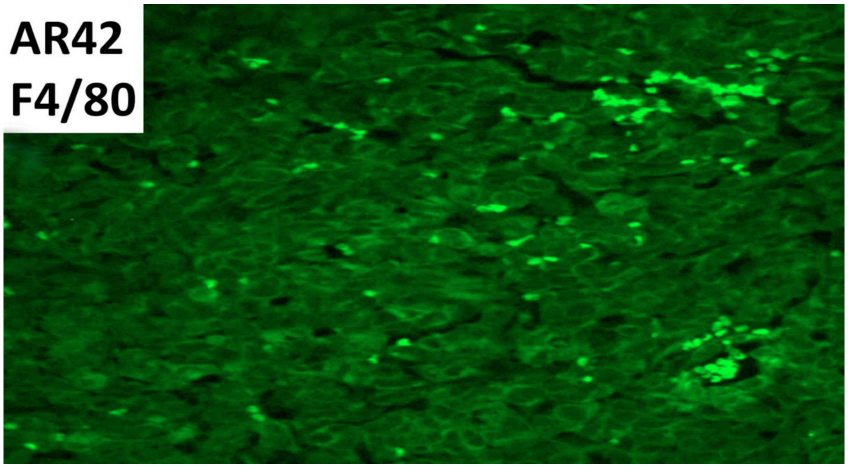

Supplementary Figure 14: Representative enlarged images of AR42 treated and [pazopanib + AR42] treated MEL28-R cells. Four micron slides of AR42 treated and [pazopanib + AR42] tumors were made and immunohistochemistry performed to determine the expression of the indicated proteins.

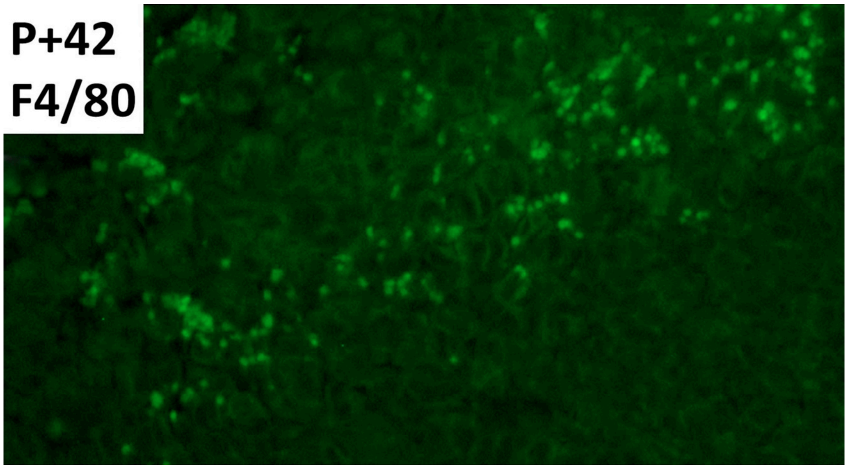

Supplementary Figure 15: Representative enlarged images of AR42 treated and [pazopanib + AR42] treated MEL28-R cells. Four micron slides of AR42 treated and [pazopanib + AR42] tumors were made and immunohistochemistry performed to determine the expression of the indicated proteins.

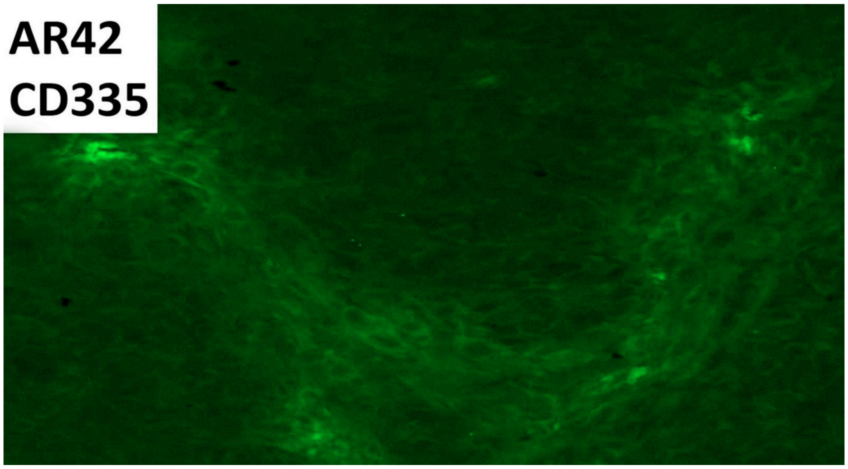

Supplementary Figure 16: Representative enlarged images of AR42 treated and [pazopanib + AR42] treated MEL28-R cells. Four micron slides of AR42 treated and [pazopanib + AR42] tumors were made and immunohistochemistry performed to determine the expression of the indicated proteins.

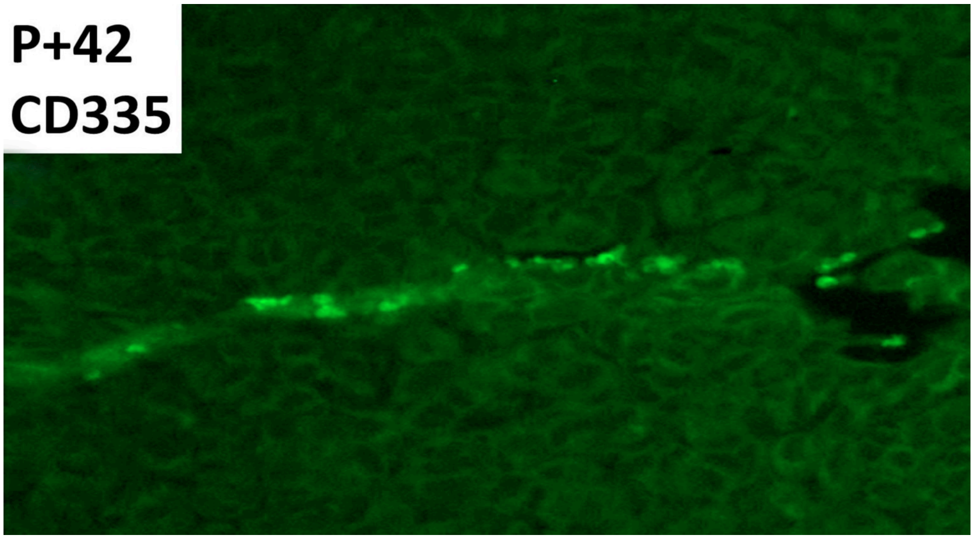

Supplementary Figure 17: Representative enlarged images of AR42 treated and [pazopanib + AR42] treated MEL28-R cells. Four micron slides of AR42 treated and [pazopanib + AR42] tumors were made and immunohistochemistry performed to determine the expression of the indicated proteins.

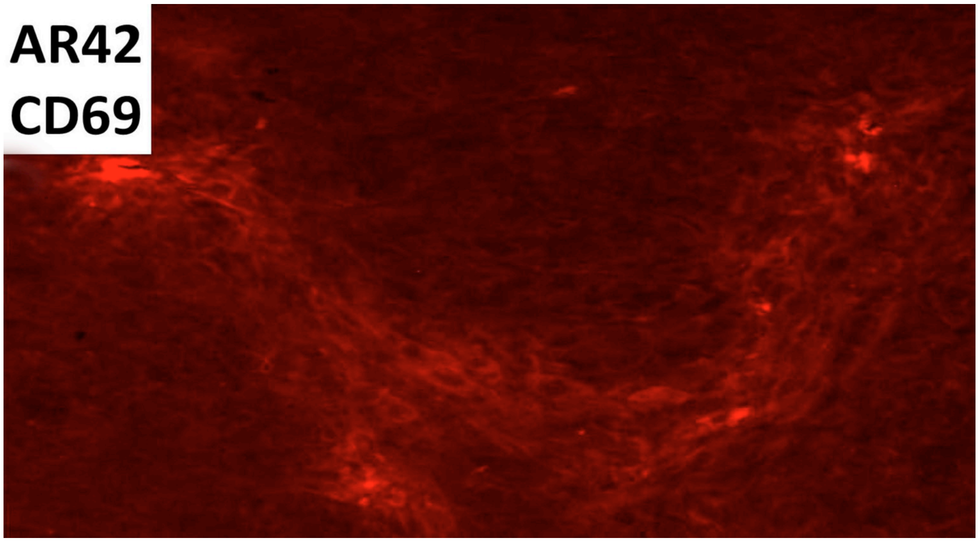

Supplementary Figure 18: Representative enlarged images of AR42 treated and [pazopanib + AR42] treated MEL28-R cells. Four micron slides of AR42 treated and [pazopanib + AR42] tumors were made and immunohistochemistry performed to determine the expression of the indicated proteins.

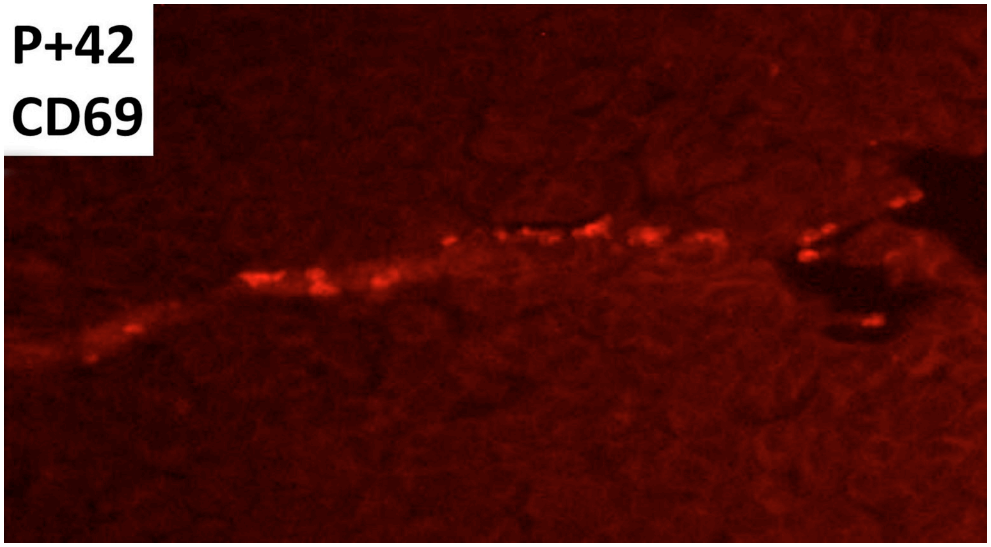

Supplementary Figure 19: Representative enlarged images of AR42 treated and [pazopanib + AR42] treated MEL28-R cells. Four micron slides of AR42 treated and [pazopanib + AR42] tumors were made and immunohistochemistry performed to determine the expression of the indicated proteins.

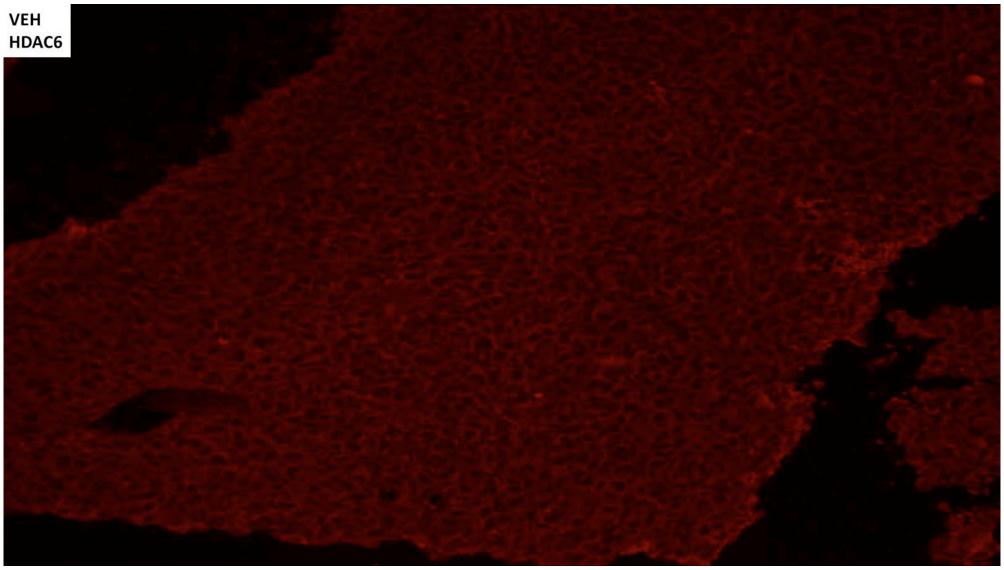

Supplementary Figure 20: Vehicle control treated tumors stained to determine HDAC6 expression. Four micron slides of vehicle treated tumors were made and immunohistochemistry performed to determine the expression of HDAC6 at 10× magnification.

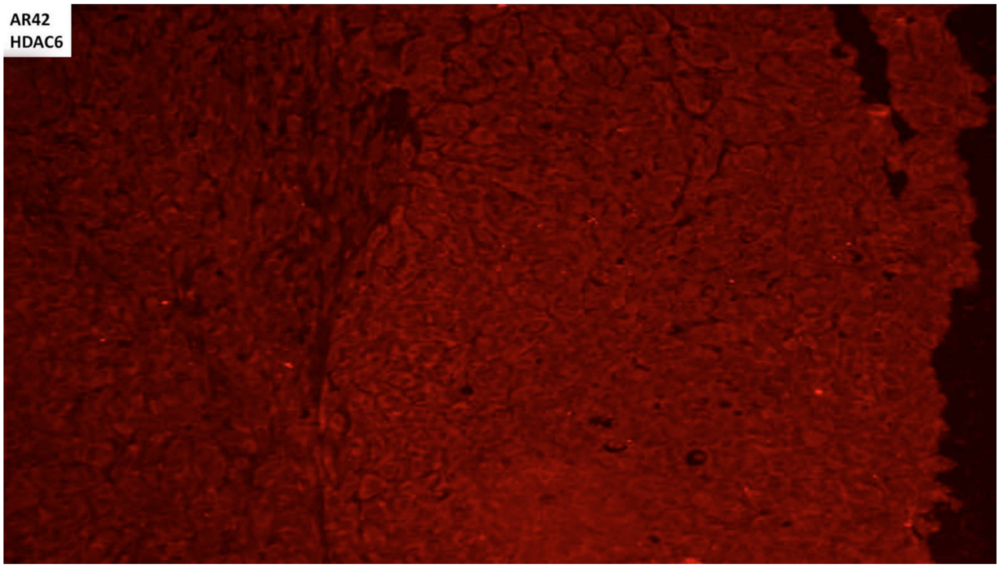

Supplementary Figure 21: AR42 treated tumors stained to determine HDAC6 expression. Four micron slides of AR42 treated tumors were made and immunohistochemistry performed to determine the expression of HDAC6 at 10× magnification.

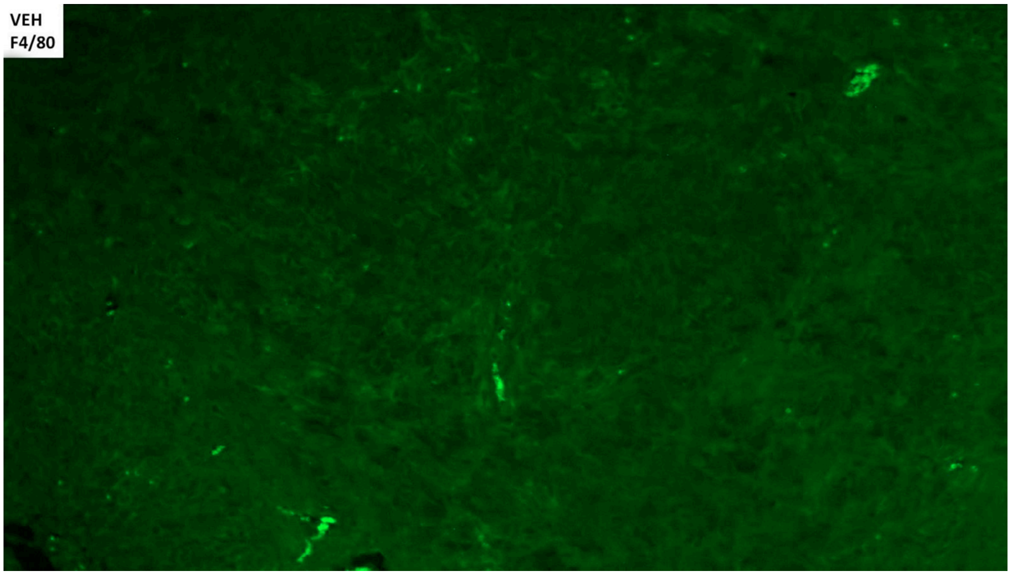

Supplementary Figure 22: Vehicle control treated tumors stained to determine F4/80 expression. Four micron slides of vehicle treated tumors were made and immunohistochemistry performed to determine the expression of F4/80 at 10× magnification.

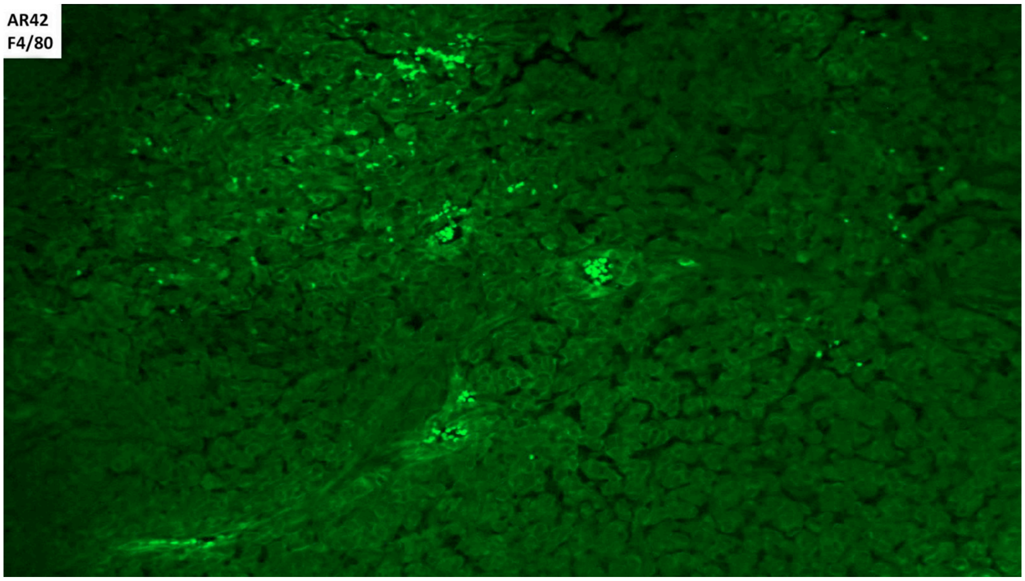

Supplementary Figure 23: AR42 treated tumors stained to determine F4/80 expression. Four micron slides of AR42 treated tumors were made and immunohistochemistry performed to determine the expression of F4/80 at 10× magnification.

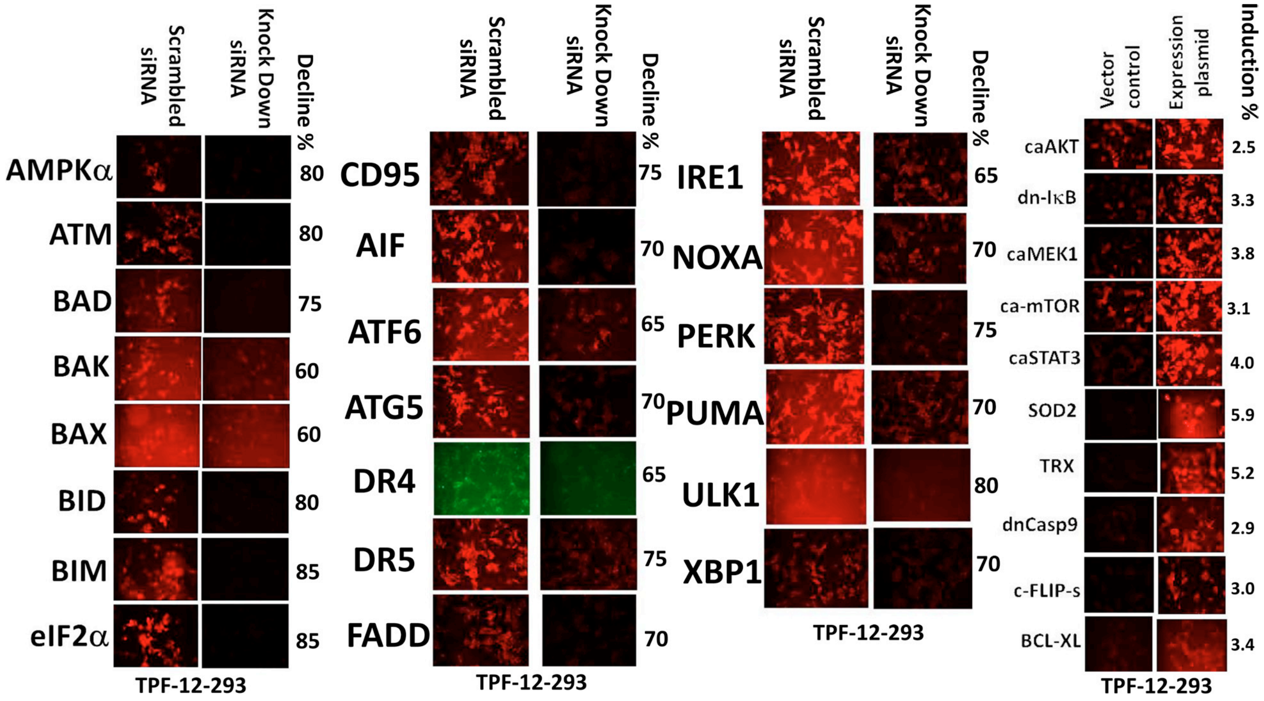

Supplementary Figure 24: Controls to show siRNA knock down of various proteins examined in the manuscript (1). The percentage decline in protein expression after siRNA exposure is presented alongside the images showing knock down. Values are rounded to the nearest 5%.

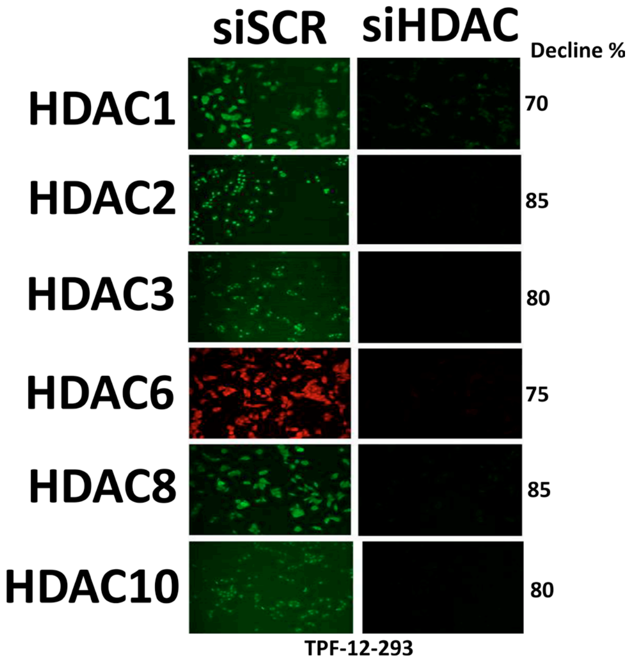

Supplementary Figure 25: Controls to show siRNA knock down of various proteins examined in the manuscript (2). The percentage decline in protein expression after siRNA exposure is presented alongside the images showing knock down. Values are rounded to the nearest 5%.
